# Tripeptide-Loaded Liposomes as Multifunctional Components in Topical Formulations

**DOI:** 10.3390/ijms26115321

**Published:** 2025-06-01

**Authors:** Michał Dymek, Maria José García-Celma, Elvira Escribano-Ferrer, Dawid Warszycki, Sławomir Kaźmierski, Łukasz Skoczylas, Małgorzata Tabaszewska, Elżbieta Sikora

**Affiliations:** 1 CUT Doctoral School, Faculty of Chemical Engineering and Technology, Cracow University of Technology, 31-155 Kraków, Poland; 2 Pharmaceutical Nanotechnology Group, Department of Pharmacy, Pharmaceutical Technology and Physical-Chemistry, University of Barcelona, E-08028 Barcelona, Spain; 3 Biopharmaceutics and Pharmacokinetics Unit, Department of Pharmacy and Pharmaceutical Technology and Physical Chemistry, Faculty of Pharmacy and Food Sciences, University of Barcelona, E-08028 Barcelona, Spain; 4 Department of Medicinal Chemistry, Maj Institute of Pharmacology, Polish Academy of Sciences, 31-343 Kraków, Poland; 5 Centre of Molecular and Macromolecular Studies, Polish Academy of Sciences, 90-363 Łódź, Poland; 6 Department of Plant Product Technology and Nutrition Hygiene, Faculty of Food Technology, University of Agriculture in Kraków, 30-149 Kraków, Poland; 7 Department of Human Nutrition and Metabolomics, Pomeranian Medical University in Szczecin, 71-460 Szczecin, Poland; 8 Faculty of Chemical Engineering and Technology, Cracow University of Technology, 31-155 Kraków, Poland

**Keywords:** bioactive tripeptides, skin permeation, liposomes, encapsulation, tyrosinase inhibitors

## Abstract

Modern dermocosmetics combine the effectiveness of active substances with the benefits of percutaneous penetration enhancers to address skin issues such as hyperpigmentation. In this study, three bioactive tripeptides (with amino acid sequences CSF, CVL, and CSN) with previously confirmed tyrosinase inhibition activity were synthesized using the solid-phase synthesis method. The structures of the obtained peptides were determined. In addition, elastase in silico and in vitro inhibition assays were carried out. The tripeptides were subsequently encapsulated into liposomes, for which key physicochemical parameters were determined, including size, zeta potential, and encapsulation efficiency. The average diameter of the prepared liposomes was approximately 100 nm across all samples. The prepared carriers were found to be stable and exhibited no cytotoxicity toward reconstructed human epidermis cells. The peptides achieved an encapsulation efficiency of approximately 20–30%, with no significant differences observed between the cationic and anionic vesicles. Liposomes containing the CSF tripeptide, which showed the strongest tyrosinase-inhibiting effect, did not transport the peptide through the human skin in an ex vivo assay to permit quantification in the receptor solution, but facilitated penetration and retention of the tripeptide within the epidermis (4.65 ± 1.81 μg/cm^2^). These findings suggest that the prepared liposomes may serve as valuable carriers of bioactive tripeptides in anti-aging cosmetics.

## 1. Introduction

Dermocosmetics, positioned at the intersection of pharmacology and cosmetology, are gaining increasing popularity among people seeking effective and safe skincare solutions. These advanced formulations are distinguished not only by the high quality of their ingredients but also by the precise selection of active substances aimed at improving both the appearance and health of the skin and hair [1,2,3]. Dermocosmetics are derived from a systemic approach, including both an in-depth understanding of skin physiology and related factors (e.g., genetics, age, gender, individual characteristics) as well as the development of modern cosmetic formulations [4,5,6]. The growing demand for dermocosmetics results from the needs of consumers to eliminate the signs of aging and more complex skin problems, such as hyperpigmentation changes [7,8,9,10], acne vulgaris [11,12,13,14], atopic dermatitis [15,16,17], microbiome disorders [18], or a tendency for allergic reactions [19,20].

The active substances contained in dermocosmetics are mainly responsible for the effectiveness of the products. To meet high consumer expectations, various ingredients are used for these purposes, including plant extracts [21], extracted single substances of herbal origin [22], thermal spring waters [18], as well as vitamins and hyaluronic acid [5]. Among the available bioactive ingredients, peptides warrant special attention as they offer a multiplicity of biological effects owing to the large number of possible combinations of amino acids. Peptides typically consist of a small number of amino acids (up to 50 residues) and are obtained both from natural sources (plants, microorganisms) and by chemical synthesis [23]. According to the diversity of their action, peptides are most often divided into four categories: (1) signal peptides [24,25], stimulating the production of selected compounds in the skin (collagen, elastin); (2) carrier peptides [26,27], providing trace elements to the skin (e.g., copper ions); (3) enzyme inhibitor peptides [28,29,30], inhibiting the activity of enzymes in the skin (e.g., elastase, collagenase, tyrosinase); and (4) neurotransmitter-affecting peptides [31], reducing the visibility of wrinkles by limiting facial muscle contractions.

Skin-lightening agents are among the most intensively researched compounds due to growing consumer demand for effective inhibitors of tyrosinase—the enzyme responsible for melanin production. Additionally, concern over the potential harmful effects of commonly used substances is rising, such as kojic acid [32,33] and hydroquinone [34], which has already been banned in certain countries. Our previous study demonstrated that peptides can serve as effective inhibitors of melanogenesis [35]. However, the hydrophilic structure of peptides poses a challenge for their penetration of the hydrophobic outer layer of the epidermis to reach melanocytes as the target site of action. To enhance their successful delivery to the deeper layers of the skin, peptides can be encapsulated within carriers such as liposomes [36]. These phospholipid-based vesicles are capable of transporting both hydrophilic and lipophilic biologically active substances, thereby offering a promising strategy for peptide release.

In this study, tripeptides with tyrosinase-inhibiting properties—CSF, CVL, and CSN—were synthesized. To investigate their potential complementary action against another enzyme associated with skin aging—elastase—molecular modeling simulations and in vitro inhibition tests were carried out. Furthermore, liposomal carriers were prepared and characterized to assess their ability to encapsulate the peptides, including their size, zeta potential, encapsulation efficiency (EE), loading capacity (LC), and irritation potential. Percutaneous penetration studies were further performed on human skin samples for the most promising carriers, with a commercially available hydrophilic copper tripeptide (GHK-Cu) serving as a reference substance.

## 2. Results and Discussion

### 2.1. Peptide Synthesis and Characterization

The details of the IR, ^1^H-NMR, ^13^C-NMR, and UV-Vis measurements are presented in the Supplementary Materials (Figures S1–S23). The ^1^H NMR spectra of the CSN, CSF, and CVL tripeptides are compared in Figure 1, with their respective amino acid assignments indicated.

**CVL (L-cysteinyl-L-Valinyl-L-leucine)**

White solid. FTIR ν [cm^−1^] 3295, 3085, 2967, 1646, 1546, 1434, 1189, 1139, 842, 800, 723.^1^H-NMR δ: 4.29 (m, 1H), 4.06 (d, 1H), 2.96 (t, 2H), 1.97 (m, 1H), 1.56 (m, 2H), 1.56 (m, 1H); 0.87 (dd, 3H), 0.83 (d, 3H), 0.77 (d, 3H). ^13^C-NMR δ: 17.72, 18.18, 20.51, 22.11, 24.33, 25.06, 29.95, 39.25, 51.44, 53.96, 59.73, 167.79, 172.95, 176.26. MS (ESI), m/z = 334.2 [M+H]^+^. M = 333.45 g/mol.

**CSF (L-cysteinyl-L-Seryl-L-Phenylalanine)**

White solid. FTIR ν [cm^−1^] 3272, 3091, 3045, 2929, 1631, 1535, 1427, 1189, 1135, 1072, 842, 800, 727, 700. ^1^H-NMR δ: 7.21 (m, 5H), 4.55 (t, 1H), 4.38 (t, 1H), 4.12 (t, 1H), 3.70 (m, 2H), 3.11 (dd, 2H), 2.90 (m, 2H). ^13^C-NMR δ: 24.90, 36.96, 54.19, 54.84, 55.47, 60.98, 127.02, 128.62, 129.28, 136.70, 167.95, 170.23, 175.54. MS (ESI), m/z = 356.4 [M+H]^+^. M = 355.41 g/mol.

**CSN (L-cysteinyl-L-Seryl-L-Asparagine)**

Creamy-white solid. FTIR ν [cm^−1^] 3293, 3083, 2962, 1660, 1533, 1430, 1191, 1135, 840, 802, 723.^1^H-NMR δ: 4.59 (t, 1H), 4.45 (t, 1H), 4.19 (t, 1H), 3.79 (t, 2H), 3.01 (m, 2H), 2.74 (m, 2H). ^13^C-NMR δ: 24.92, 36.39, 50.06, 54.19, 55.58, 60.87, 168.15, 170.63, 174.47, 174.87. MS (ESI), m/z = 323.2 [M+H]^+^. M = 322.34 g/mol.

### 2.2. Elastase Inhibition Assay: In Silico and In Vitro Studies

In a previous study [35], it was demonstrated that the developed peptides act as inhibitors of tyrosinase, an enzyme whose dysregulated activity contributes to the formation of skin discolorations [37]. Other enzymes present in the body, such as elastase, can also negatively affect skin appearance. Elastase is a proteolytic enzyme that plays a critical role in skin aging processes. Its primary function is to degrade elastin, a protein responsible for the skin’s elasticity and resilience. Over time, and under the influence of external factors such as UV radiation and environmental pollution, elastase activity increases. This leads to elastin degradation, resulting in the skin losing firmness, developing wrinkles, and becoming saggy [38].

Given this background, as a complement to tests for anti-discoloration activity, this study investigated whether synthesized peptides might exhibit a broader range of effects by also inhibiting elastase.

To assess whether the tested tripeptides could potentially bind to elastase’s active site, preliminary molecular simulation studies were conducted. This low-cost method allows for the rapid screening of multiple compounds and the in silico evaluation of their binding potential. After preparing the ligands and elastase (PDB ID: 1BRU), a docking study was performed, with the results summarized in Table 1.

**Figure 2:**
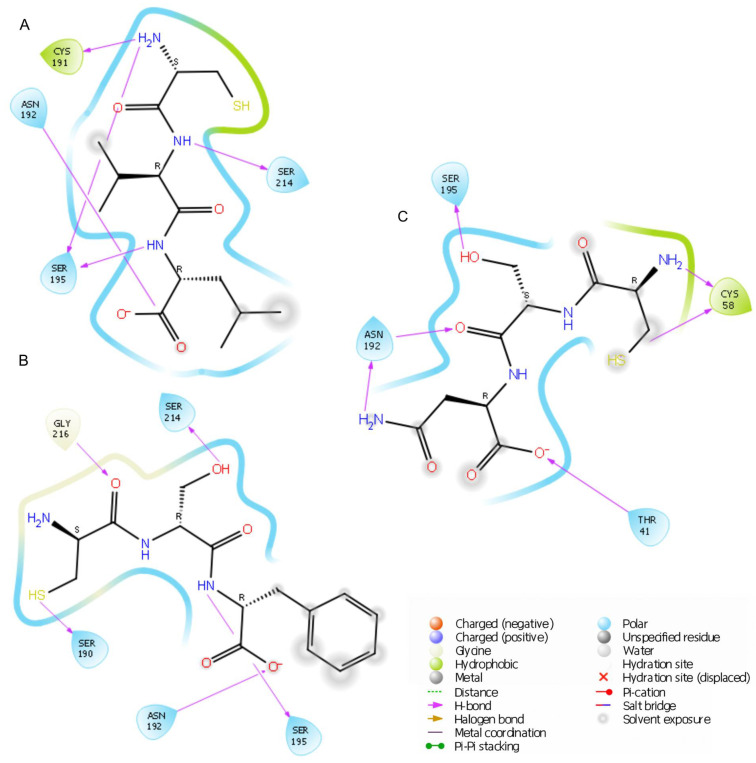
Visualization of binding interactions for the tripeptides CVL (**A**), CSF (**B**), and CSN (**C**) within the elastase binding site of 1BRU.

The lowest docking score was obtained for the tripeptide CSF (−6.149 kcal/mol), followed by CSN (−5.304 kcal/mol) and CVL (−4.755 kcal/mol). Superposition analysis indicated that the CSF and CVL peptides adopt similar orientations on the enzyme’s surface. In contrast, CSN positions its N-terminus in a region characterized by higher electrostatic potential values (Figure S24 in Supplementary Materials). A more detailed understanding is provided by the in-depth analysis of interaction types (Figure 2) within the binding pocket.

All simulated tripeptides showed a similar binding mode and formed hydrogen bonds with Asn192 and Ser195. CSF, the peptide with the lowest docking score, established hydrogen bonds with Ser214 via the hydroxyl group of serine (2.07 Å), with Gly216 (3.25 Å) via the carbonyl oxygen of cysteine, and with Ser190 through the thiol group. Two additional hydrogen bonds were observed with Ser195 and Asn192 (via the serine oxygen and the amine group, at 1.86 Å and 1.79 Å, respectively).

The binding mode of the CVL tripeptide revealed the presence of five hydrogen bonds: Cys191 and the amine group of cysteine (2.67 Å), Asn192 and the oxygen atom of leucine (1.79 Å), Ser214 and the amine group of valine (2.88 Å), as well as two interactions involving Ser195 with the cysteine amine group and leucine amine group (1.93 Å and 2.21 Å, respectively). Finally, six hydrogen bonds were identified for the CSN peptide. Double interactions were noted with enzymatic residues Cys58 (2.66 Å and 2.48 Å) and Asn192 (2.07 Å and 2.20 Å). Additionally, hydrogen bonds were formed with Thr41 (1.74 Å) and Ser195 (1.87 Å).

A previous study indicated that effective inhibitors often form hydrogen bonds with Asn192 and Ser195 [39]. Interactions with Gly216 have also been observed, as exemplified by caffeine [40]. Aleosin interacts with Cys58, similar to CSN [41]. These amino acid residues are key components of the binding pocket in elastase. The formation of similar interactions by the peptides developed in this study, CVL, CSF, and CSN, highlights the conserved binding mechanism of elastase and the universal nature of these contacts. This suggests that these peptides are likely to function as active inhibitors.

To verify the in silico findings, in vitro tests were carried out using elastase from the porcine pancreas. The final concentration of each peptide during the test was 714 μM. As shown in Table 1, CVL had the highest elastase inhibition activity (35.3 ± 8.6%), followed by CSF (27.6 ± 1.2%) and CSN (28.9 ± 7.5%). However, the differences in inhibition between the peptides were not statistically significant (*p* > 0.05). The similarity in inhibition levels among the peptides might be related to their comparable chemical structures, as all of the compounds are tripeptides with cysteine at the N-terminus. This was also reflected in the docking results and confirmed by interaction analysis (Figure 2), where all peptides formed hydrogen bonds with Asn192 and Ser195.

In the previous study, the peptides were confirmed to effectively reduce melanogenesis by modulating tyrosinase activity [35]. The confirmation of their elastase inhibition activity in the present study can be seen as a complementary property that further supports the anti-aging potential of these peptides for application in modern dermocosmetics. Given the large number of possible peptide chain modifications, the activity of these compounds can be altered and studied in further research.

### 2.3. Physicochemical Properties and Cytotoxicity of Peptide-Loaded Liposomes

**Figure 3:**
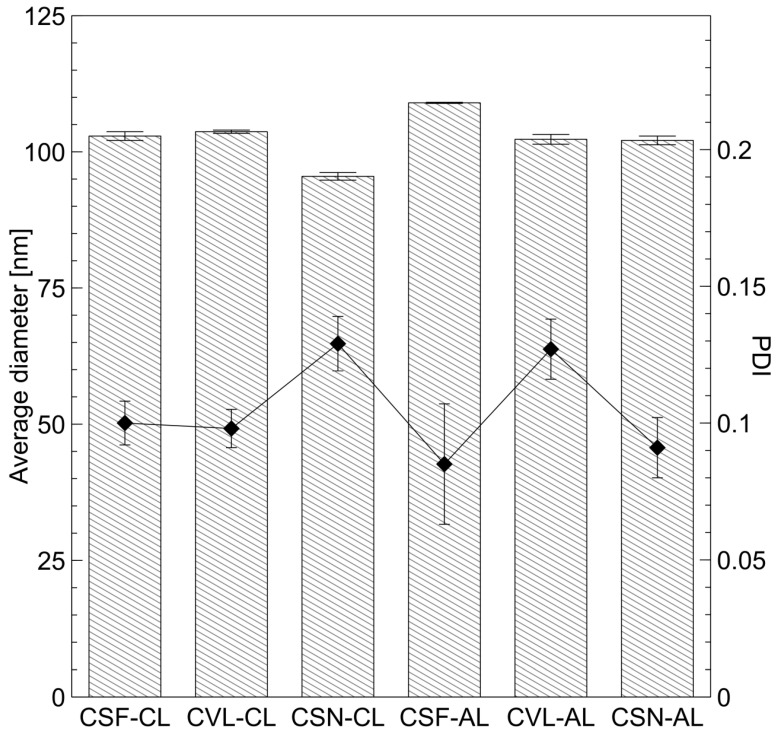
Size analysis of cationic liposomes (CLs) and anionic liposomes (ALs) containing the synthesized tripeptides. The dashed bars correspond to the left-side axis (average diameter) and black squares correspond to the right-side axis (polydispersity index, PDI). All values are expressed as mean ± SD.

In this study, anionic and cationic liposomes encapsulating the synthesized peptides were prepared. DCP (dicetyl phosphate) and SA (stearylamine) were used to introduce anionic and cationic charges to the vesicle surface, respectively. The proportions of lipids used—7% SA for CLs (cationic liposomes) and 15% DCP for ALs (anionic liposomes)—were identified as optimal in a previous study [42]. ALs containing the commercially available copper peptide GHK-Cu served as a comparative reference. Key physicochemical properties, including size, zeta potential, and cytotoxicity, were determined for the prepared vesicles. The average diameter and PDI (polydispersity index) of the liposomes are presented in Figure 3. As all liposomal formulations demonstrated relatively narrow and unimodal size distributions, the values were calculated as the arithmetic mean of three independent measurements.

Analysis of the data confirmed the formation of large unilamellar vesicles (LUVs). The diameter of all carriers, regardless of surface charge or encapsulated tripeptide, was approximately 100 nm, and all vesicles exhibited narrow polydispersity indices (~0.1). This uniformity is attributed to the standardized preparation technique combined with extrusion through a polycarbonate membrane with 100 nm pores. This method provided precise control over carrier production, ensuring high reproducibility and uniformity [43]. The extrusion process was specifically chosen to yield vesicles with favorable characteristics for dermal delivery, such as sufficient encapsulation volume, stability, and compatibility with hydrophilic peptides. Importantly, the goal of this study was not to achieve systemic delivery via the transdermal route, but rather to deliver the encapsulated peptides into the viable layers of the skin, where melanocytes—the intended target of the anti-hyperpigmentation peptides—reside.

The lack of significant variation in physical parameters among liposomes containing different peptides can be attributed to the peptides’ similar structural characteristics. This includes comparable molar masses and the shared presence of cysteine at the N-terminus. The anionic formulations (CSF-AL, CVL-AL, CSN-AL) exhibited closely aligned zeta potential values, ranging from approximately −35 to −39 mV, with no statistically significant differences between them (Table 2). These values were comparable to the zeta potential of the empty anionic liposomes (−38.6 ± 1.3 mV). This uniformity suggests that, under identical preparation conditions and with the same lipid composition, the encapsulated tripeptide does not notably influence the surface charge of anionic liposomes.

In contrast, within the cationic series, although CSF-CL and CVL-CL showed similar zeta potentials (+34.2 mV and +36.0 mV, respectively), the CSN-CL formulation stood out with a significantly higher value (+44.7 mV), while the zeta potential of empty cationic liposomes was +42.7 ± 2.5 mV. Since all formulations were prepared using the same method and contain structurally related peptides, this difference likely stems from subtle variations in peptide–lipid interactions. The slightly lower zeta potential of the CSF- and CVL-encapsulated liposomes may indicate a mild interaction and adhesion of the peptides to the liposome surface. Notably, all tested liposomes—regardless of charge—demonstrated high absolute zeta potential values (above 30 mV), a characteristic commonly associated with strong electrostatic repulsion. This repulsion plays a key role in preventing particle aggregation and contributes to the colloidal stability of the systems during storage and handling. The elevated zeta potential observed for CSN-CL may therefore reflect a marginally stronger repulsive interaction, enhancing its physical stability relative to the other cationic formulations.

Although the preparation method is highly standardized and reproducible, small differences in peptide behavior—whether due to interaction with the lipid bilayer or its spatial distribution within the vesicle—can lead to measurable shifts in surface charge. These findings highlight the sensitivity of zeta potential to even minor molecular differences, underscoring the need to consider peptide-specific effects even in tightly controlled formulations.

**Figure 4:**
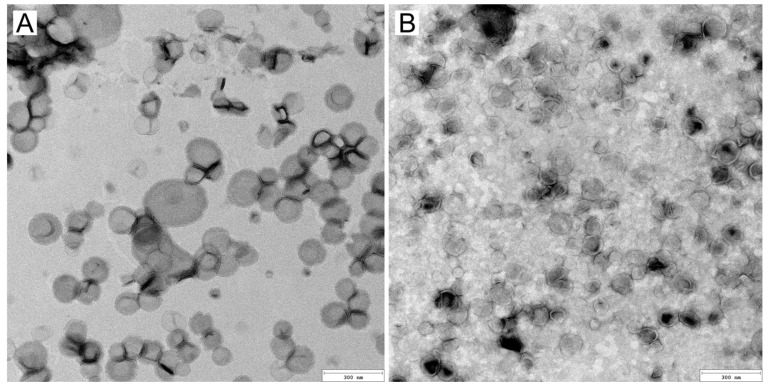
TEM images of CSF-CL (**A**) and CSF-AL (**B**) liposomes. The bar represents 300 nm.

Figure 4 presents the results of TEM imaging. While limited to structural analysis in the dry state, TEM provides crucial complementary information to DLS analyses. All liposomes exhibited a similar size and shape, with no agglomerates that could affect sample stability or the presence of non-liposomal particles (e.g., impurities or undissolved lipids). The high homogeneity of the samples is again attributed to the preparation method used, which facilitated size reduction and uniformity. The liposomes maintained their structural integrity, showing no deformation or asymmetry. They retained their morphology even after sample preparation for TEM, further demonstrating high mechanical stability. TEM analysis confirmed the presence of a single lipid bilayer, consistent with their classification as LUVs. Future application of advanced techniques such as Cryo-TEM could provide additional confirmation of these findings and insights into the lipid dynamics in an aqueous environment.

Another critical parameter describing liposome properties is the EE (Table 2). This ratio, representing the concentration of peptide encapsulated in the carrier relative to the free substance in the surrounding solution, is vital for factors such as percutaneous penetration efficiency, bioavailability, and active ingredient stability in formulations. As shown in Table 2, there were no statistically significant differences in EE between the cationic and anionic liposomes containing the synthesized tripeptides (*p* > 0.05). It is also reflected in the loading capacity (LC), which is expressed as the mass of encapsulated peptide divided by the total mass of lipids used for liposome preparation. Given that the lipid concentration was consistent across all samples (25 mg/cm^3^), these results correlate with the EE values, with the highest values observed for cationic CSF liposomes (0.68 ± 0.14 mg/mg) and anionic CSF liposomes (0.59 ± 0.14 mg/mg).

Given their structural similarity, the CVL, CSF, and CSN tripeptides have comparable isoelectric points (pI), at approximately 5.33, as calculated using the tool provided by Kozlowski [44]. Under the liposome production conditions (pH = 6), the peptides are anionic, but the pH is only slightly above the pI. Consequently, while the peptides exist in an anionic form, their net charge remains relatively low. This limited ionization is insufficient to cause significant electrostatic attraction to the CLs or repulsion from the ALs. Furthermore, the synthesized peptides are hydrophilic, with logP values [45] of −0.13, −0.92, and −3.37 for CVL, CSF, and CSN, respectively, and share a similar molar mass and size. Additionally, some studies show that the cysteine residue, which is present in all tested peptides, can interact with the polar lipid head groups on the liposome surface [46]. These findings suggest that the comparable values of the peptides EE are caused by their structural similarity. The chosen encapsulation method—extrusion combined with freeze–thaw cycles—also contributed to the stability and uniformity of the liposomes.

**Figure 5:**
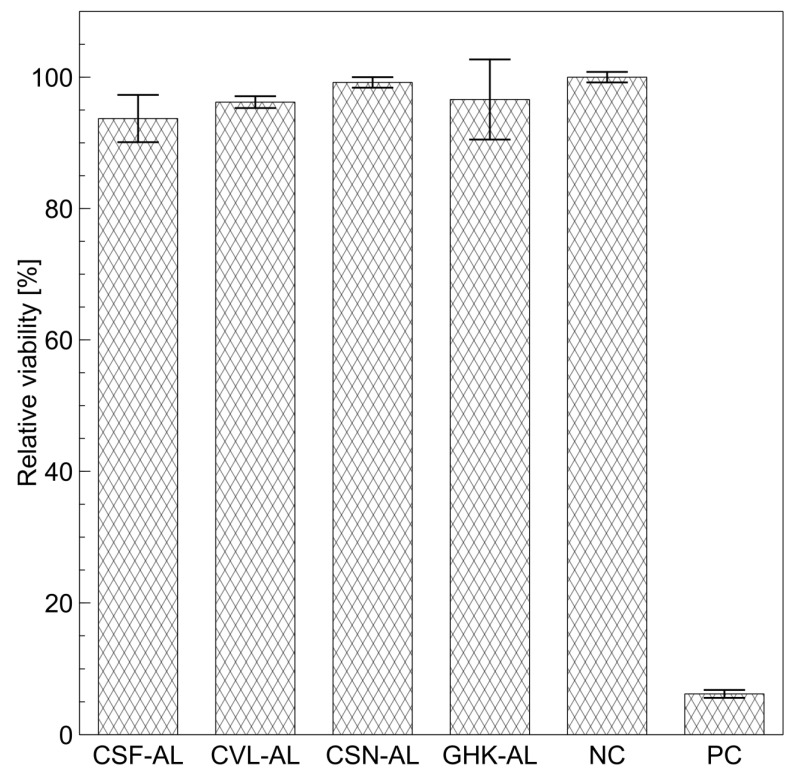
Relative viability (RV) for anionic liposomes containing synthesized peptides (CSF-AL, CVL-AL, CSN-AL) or a commercially available copper tripeptide (GHK-AL). Phosphate buffer pH 7.4 was used as the negative control (NC), and sodium dodecyl sulfate served as the positive control (PC). Values are shown as mean ± SD.

The determination of relative viability (RV) is a crucial aspect of a cytotoxicity evaluation for chemicals and formulations such as liposomes. RV provides insights into the degree to which a tested substance affects cell survival relative to controls, facilitating a safety assessment and evaluation of potential biological applications [47,48]. Given the similar EE observed across all carriers, only ALs were selected for subsequent experiments. This is because cationic vesicles, while effective, are more commonly associated with potential skin irritation [49]. The cytotoxicity test results for peptide-loaded liposomes are presented in Figure 5.

All tested liposome samples exhibited high RV values, close to 100%, when tested on reconstructed human epidermis (RHE) cells, indicating their non-cytotoxic nature. These RV values were comparable to those of the control sample (phosphate buffer), suggesting that the liposomes are non-toxic for dermatological applications. In contrast, sodium dodecyl sulfate, used as a positive control, demonstrated a very low RV (6.2%). Furthermore, the results suggested that the liposome preparation process did not introduce contaminants or artifacts such as chloroform residues used during the creation of the lipid film, which could negatively impact cell viability. Previously, we evaluated the cytotoxicity of the crude peptide solutions [35]. When comparing these results to the corresponding liposomal formulations, we observed no significant change in cytotoxicity for CSN-AL liposomes, which is consistent with our earlier findings showing that the CSN peptide alone exhibited the highest cell viability, consistently close to 100%. Similarly, the CSF-loaded liposomes did not show any notable difference in cytotoxicity, likely due to the inherently low cytotoxicity of the unencapsulated CSF peptide. Interestingly, for the CVL peptide, encapsulation in liposomes led to a marked improvement in cell viability, increasing from 77.8 ± 19.9% to 96.2 ± 0.9%, along with a significant reduction in variability. These findings suggest that the use of liposomes may also be advantageous from a cytotoxicity perspective, possibly due to the protective effect of phospholipids on the cells.

### 2.4. Permeation Studies

The final evaluation of the prepared carriers involved percutaneous penetration tests carried out in Franz diffusion cells using human skin samples. Given the comparable EE and RV across all tested liposomes, the CSF tripeptide encapsulated in anionic carriers was selected for further testing owing to its superior tyrosinase-inhibiting properties. For comparison, CSF was also tested at the same concentration in a 50% aqueous solution of Transcutol. A commercially available copper peptide (GHK-Cu), both in aqueous solution and in liposomes, served as a reference for this evaluation. Samples of the receptor solution were collected over 24 h, and the peptide content was analyzed chromatographically (HPLC).

**Figure 6:**
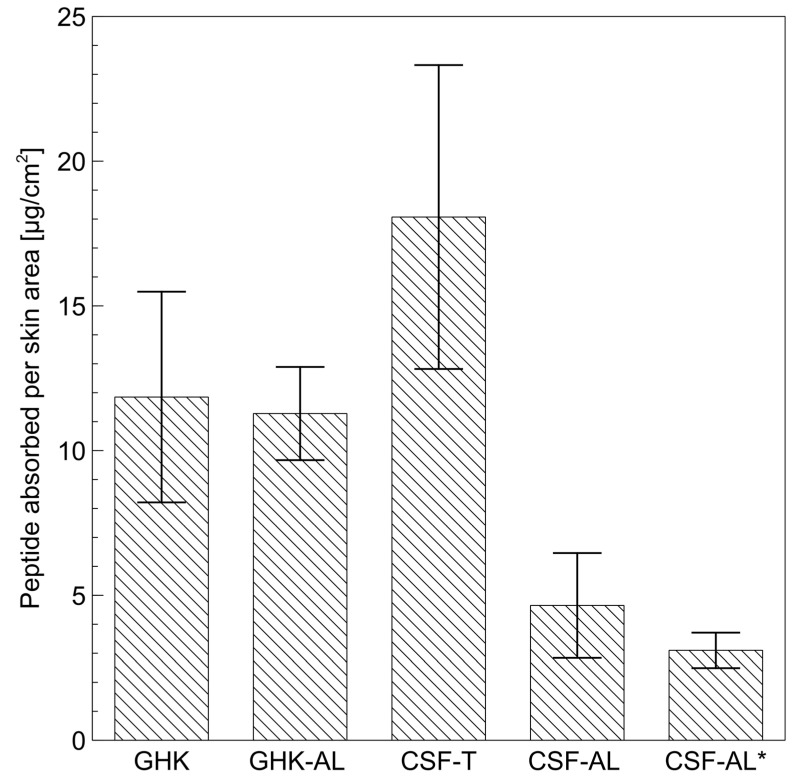
Peptide penetration per cm^2^ of human skin after 24 h for a copper tripeptide solution (GHK) on its own or encapsulated in anionic liposomes (GHK-AL) and CSF tripeptide in 50% Transcutol^®^ (CSF-T) or encapsulated in anionic liposomes (CSF-AL). The asterisk (*) indicates the peptide retained in Strat-M^®^ Membranes. Values are expressed as the mean ± SD.

No peptides were detected in the receiver solution, indicating that none of the tested systems were absorbed across the skin during the experiment. The SC represents a protective barrier against external factors [50], and due to its brick-and-mortar structure and lipophilic nature, hinders the penetration of hydrophilic active ingredients such as the tested CSF and GHK-Cu peptides, even when delivered via liposomes. However, liposomes can interact with skin lipids and release encapsulated ingredients into the skin [51], where they can bind electrostatically or through hydrogen bonding. Importantly, for skin-lightening formulations, it is more critical to deliver peptides into the skin (penetrate) rather than have them absorbed to reach the receptor fluid, as the melanocytes responsible for melanin production reside within the epidermis [52]. To quantify the extent of peptide penetration, skin samples were rinsed to remove formulation residues, sectioned, and subjected to 24 h extraction. The peptide amounts retained in the skin were normalized to the skin area (cm^2^), and the results are shown in Figure 6.

The liposomally encapsulated peptide (GHK-AL) achieved similar retention in the skin (11.28 ± 1.61 μg/cm^2^) compared to its free form GHK-Cu (11.85 ± 3.64 μg/cm^2^); this difference is not statistically significant. This suggests that liposomes do not significantly enhance the absorption of the GHK peptide into the skin. This may be due to the small size of the peptide, which is capable of penetrating the stratum corneum on its own [53]. The GHK-Cu tripeptide has been previously described in the literature as being able to penetrate the skin. Alternatively, the peptide may also be released from the carrier on the surface of the skin instead of being transported into the skin by liposomes. Nevertheless, liposomes may be used as a stabilizing agent for the peptide while also providing cytoprotective benefits for the cells.

Among all the tested samples, the CSF peptide delivered in a 50% Transcutol solution (CSF-T) exhibited the highest retention in the skin (18.07 ± 5.25 μg/cm^2^). Transcutol is a well-documented penetration enhancer [54,55,56], acting as a solubilizer and plasticizer for SC lipids, facilitating the passage of hydrophilic substances through the skin barrier. The sample, including this penetration enhancer, thus served as a positive control model for penetration, providing a benchmark to evaluate other carriers. In comparison, liposomally encapsulated CSF (CSF-AL) achieved significantly lower retention (4.65 ± 1.81 μg/cm^2^), likely due to the direct effect of Transcutol on skin properties, increasing the degree of retention in the skin [54]. Liposomes interact and fuse with skin lipids to deliver the peptide. The liposome must first integrate into the lipid layer to then release the encapsulated peptide. This process facilitates a gradual release process, maintaining a consistent active substance concentration and prolonging its action. It is worth noting that the tested liposomes had an EE of 29.65 ± 6.91%, meaning that up to 70–75% of the peptide was present in the surrounding solution, which significantly limits its potential for skin penetration. This factor reduced the difference between CSF-T and CSF-AL. Achieving a higher EE may be challenging due to the hydrophilic nature of the peptide, which favors the aqueous phase surrounding the liposomes. Modifying the liposome structure could be an alternative strategy to enhance encapsulation and delivery efficiency.

A previous study demonstrated that ALs based on DCP exhibited higher fluorescence anisotropy values [42], indicating a more rigid lipid bilayer compared to CLs containing SA. The increased bilayer stiffness may enhance stability; however, this property could also impede the ability of liposomes to penetrate the skin layers effectively. Developing more flexible, deformable liposomes as carriers for CSF may therefore represent a promising approach for optimizing biocompatible delivery systems for our bioactive peptides. The concept of using deformable liposomes as advanced carriers to enhance skin penetration of active compounds is well known and thoroughly described in the literature [57,58,59]. These systems are specifically engineered to possess greater membrane flexibility than conventional liposomes, allowing them to more effectively traverse the stratum corneum. Ethosomes, for example, utilize high concentrations of ethanol to increase bilayer fluidity. Transfersomes incorporate edge activators—typically surfactants—that disrupt the lipid bilayer to enhance deformability. Invasomes combine phospholipids, ethanol, and terpenes to improve flexibility and skin permeation, while flexosomes are another example of soft nanocarriers designed for optimized transdermal delivery through modulation of membrane rigidity. These deformable vesicles are able to pass through the narrow intercellular spaces of the stratum corneum, leading to improved transport of encapsulated compounds into and across the skin.

In this study, conventional liposomes were selected as a model delivery platform for the CSF peptide. However, such systems can potentially be further optimized to improve their interaction with the skin barrier.

Additionally, we evaluated the penetration of CSF-loaded liposomes using the synthetic Strat-M^®^ membrane, promoted as an alternative to human skin testing [60]. As shown in Figure 6, peptide retention in Strat-M^®^ (CSF-AL *) was similar (3.10 ± 0.61 μg/cm^2^) to that in ex vivo human skin (CSF-AL; 4.65 ± 1.81 μg/cm^2^), and no peptide reached the receptor medium. The differences between the samples were not statistically significant. These findings underscore that Strat-M^®^ is valuable for initial formulation screening. However, definitive assessment of skin delivery—especially for hydrophilic peptides—requires complementary studies using excised human or animal skin to capture both intercellular and appendageal transport mechanisms. While Strat-M^®^ offers excellent homogeneity and ease of use, it does not include anatomical features such as hair follicles or sweat ducts, which in vivo can constitute important routes (skin appendages) for the entry of macromolecules and peptides into the epidermis.

## 3. Materials and Methods

### 3.1. Materials

The copper tripeptide (SpecPed-GCu11P, Spec-Chem Industry Inc., Nanjing, China), intended for cosmetic use, and 1.0–1.5 MDa Medium Molecular Weight Sodium Hyaluronate (Shandong Focusfreda Biotech Co., Ltd., Qufu, China) were generously provided by Alfa Sagittarius (Kraków, Poland). Fmoc-protected amino acids (Cys, Ser, Phe, Val, Asn, Leu), along with tri-isopropylsilane (TIPS), 1-dodecanethiol (NDM), ethylcyano(hydroxyimino)acetate (Oxyma Pure), 4-dimethylaminopyridine (DMAP), N,N′-di-isopropylcarbodiimide (DIC), trifluoroacetic acid (TFA), tri-isopropylsilane (TIPS), Tween^TM^ 80 (Polysorbate 20), N-succinyl-Ala-Ala-Ala-p-nitroanilide, elastase from the porcine pancreas, Strat-M^®^ Membranes, and HMPB ChemMatrix^®^ resin were all sourced from Merck (Darmstadt, Germany). Hydrogenated lecithin (HL, Emulmetik 950, Lucas Meyer Cosmetics, Massy, France) was supplied by Naturallia Sp. z o.o. (Brzeg, Poland). Transcutol^®^ P (diethylene glycol monoethyl ether) was from Gattefossé (Saint-Priest, France). Stearylamine (SA, 97%), dicetyl phosphate (DCP), and cholesterol (ChL, 95%) were obtained from Alfa Aesar (Thermo Fisher (Kandel) GmbH, Kandel, Germany). Tris-HCl buffer was purchased from Pol-Aura (Zawroty, Poland). Strat-M^®^ membrane was purchased from Millipore (Billerica, MA, USA), and the dermatomed human skin sheet (0.4 mm thickness) was purchased from Biopredic International (Saint-Grégoire, France). A Spectra/Por 1 regenerated cellulose membrane, with a 6–8 kDa molecular weight cut-off (MWCO) for microdialysis tests, was purchased from Spectrum Laboratories Inc. (Breda, The Netherlands). PP reactors with PE Frit, used for peptide synthesis, were acquired from Carl Roth GmbH+Co (Karlsruhe, Germany). Other reagents, including potassium dihydrogen phosphate (Avantor Performance Materials Poland S.A., formerly POCH S.A., Gliwice, Poland), sodium hydrogen phosphate (Avantor), methanol (Chempur, Piekary Śląskie, Poland), diethyl ether (Chempur), and acetonitrile (Chempur), were of analytical grade. Deionized water for all formulations was further purified using a Milli-Q system.

### 3.2. Peptide Synthesis and Characterization

#### 3.2.1. Peptide Solid Phase Synthesis (PSPS)

To obtain the desired peptides (CSF, CVL, CSN), solid-phase synthesis was carried out using a previously reported procedure [61] with minor modifications, including the use of Oxyma Pure as a safer alternative to HOBt. Briefly, HMPB ChemMatrix^®^ resin (0.47 mmol/g loading) was soaked for 30 min in DMF. To couple the first amino acid, 5 equivalents of Fmoc-protected amino acid in DMF, 5 equivalents of DIC, and 0.1 equivalent of DMAP in DMF were used relative to the resin. The coupling reaction for the first amino acid was conducted in a syringe with a frit at room temperature (20–25 °C) for 3 h. To ensure complete acetylation of unreacted OH groups, 2 equivalents of acetic anhydride and DIPEA relative to the resin were added and mixed for 30 min. The resin was washed three times with DMF after each amino acid coupling step. Fmoc deprotection was carried out using a 20% piperidine solution in DMF, washing the resin five times with DMF each time, followed by adding solutions of the next amino acid, Oxyma Pure, and DIC in DMF. Final cleavage of the peptide from the resin was carried out using a mixture of (*v*/*v*) 87.1% TFA, 4.3% TIS, 4.3% NDM, and 4.3% water for 2 h. The reaction mixture was then expelled from the syringe into cold diethyl ether, centrifuged, and the resulting precipitate was washed three times with ether.

#### 3.2.2. Structural Characterization of Peptides

The synthesized peptides were characterized using various techniques to confirm the desired structures. ^1^H-nuclear magnetic resonance (NMR) and ^13^C-NMR measurements were performed on a Bruker Avance III 500 spectrometer (Bruker BioSpin, Rheinstetten, Germany), operating at a frequency of 500.13 MHz for ^1^H and 125.16 for ^13^C, equipped with a GAB/2 gradient unit capable of producing B0 gradients with a maximum strength of 50 G/cm. An automated tuned and matched (ATMA) 5 mm triple-channel TBO (BB/H-F/D) probe head with actively shielded Z-gradients coil was utilized. During all measurements, the temperature was controlled and stabilized with a BCU 05 cooling unit managed by a BVT3200 variable temperature unit. All spectra were recorded at 295 K using 5 mm NMR tubes. For each measurement, the sample was thermostated for 5 min before data accumulation. All spectra were acquired, processed, and plotted using the TopSpin 3.5 (pl6) program (Bruker) running on a PC using Windows 7 Professional. For 1D ^1^H spectra, between 32 and 64 scans were accumulated per FID of 64K data points with a 1 s relaxation delay (D1). The spectral width was set to 8012 Hz (16 ppm), resulting in an acquisition time of 3.99 s (AQ). The original pulse program, zg30, was used. FIDs were zero-filled twice and apodized with the LB function (0.3 Hz) prior to the Fourier transformation. Then, 1H spectra with water signal suppression were recorded using excitation sculpting with a gradient pulse sequence. Before running the suppressed spectra, the shift of the water signal and the 1H π/2 pulse length were checked and corrected. 1D ^13^C spectra were recorded using the zgpg30 pulse program with 64K data points of FIDs and 2 s for relaxation delay and waltz-16 sequence for decoupling. Between 2048 and 5120 scans were accumulated for each spectrum. FIDs were zero-filled twice and apodized with the LB function (2 Hz) prior to the Fourier transformation. For 2D spectra, except HMBC, original Bruker pulse programs (versions with pulse field gradients) were used. Spectral parameters were usually as follows:

COSY: data matrix (F2xF1): 4096 × 256 with 12 scans per experiment and a relaxation delay of 1 s. Fourier transformation was used in the final 2048 × 1024 data points matrix with a QSINE = 0 apodization function in both dimensions.

^1^H-^13^C HSQC-edited version: data matrix (F2 × F1): 4096 × 256 with 20 scans per experiment and relaxation delay of 1.5 s. Fourier transformation was used in the final 2048 × 1024 data points matrix with a QSINE = 2 apodization function in both dimensions.

^1^H-^15^N HSQC: data matrix (F2 × F1): 2048 × 64 with 24 scans per experiment and a relaxation delay of 1.5 s. Fourier transformation was used in the final 2048 × 1024 data points matrix with a QSINE = 2 apodization function in both dimensions.

For ^1^H-^13^C HMBC spectra, the Impact-HMBC pulse program was used with the following spectral parameters: data matrix (F2 × F1): 2048 × 256 with 20 scans per experiment and a relaxation delay of 0.2 s. Fourier transformation was used in the final 2048 × 1024 data points matrix with a QSINE = 2 apodization function in both dimensions.

Mass spectrometry with electrospray ionization was conducted on a SCIEX 4500 TripleQuad system (SCIEX, Framingham, MA, USA). Additionally, ultraviolet–visible (UV-Vis) spectra for the aqueous solution of the active ingredient were obtained with a Nanocolor UV/Vis spectrophotometer (Macherey-Nagel, Dueren, Germany) using a 10 mm path length quartz cuvette, across a wavelength range of 190–800 nm. Fourier-transform infrared (FTIR) spectra were recorded with a Nicolet iS10 FT-IR spectrometer (Thermo Fisher Scientific Inc., Waltham, MA, USA) equipped with an MCT detector. The results of the structural characterization are shown in the Supplementary Materials (Figures S1–S18).

### 3.3. In Silico Elastase Inhibition Study

The three-dimensional model of elastase (PDB ID: 1BRU) was prepared using the Protein Preparation Wizard tool in Maestro (Schrödinger Release 2024-4, Schrödinger, New York, NY, USA) [62]. Initial pre-processing steps included removing the elastase inhibitor, 2-(2-hydroxy-cyclopentyl)-pent-4-enal. Bond orders were then assigned, hydrogen atoms added, and both zero-order metal and disulfide bonds were generated. Missing side chains and loop regions were completed using Prime [63,64]. For pH 7.4, Epik (Schrödinger) [65,66] was used to generate heteroatom states. The receptor grid was then prepared using Glide [67,68,69,70] (Schrödinger), centered on the elastase inhibitor’s binding site, with default parameters: van der Waals radius scaling factor set to 1.0, a partial charge cutoff of 0.25, and no additional constraints.

All studied peptides (CSF, CVL, CSN, GHK) were processed using the LigPrep tool in Maestro software (Schrödinger Release 2024-4, Schrödinger). The three-dimensional structures of the test compounds were optimized to generate their ionized forms at pH = 7.4, utilizing the OPLS4 force field [71]. Ligands were docked to elastase with default settings (standard precision, flexible ligand sampling, nitrogen inversion and ring conformation sampling, targeted sampling of torsions for all specified functional groups, and added Epik state penalties to the docking score). After docking, minimization was performed for five poses per ligand.

### 3.4. Elastase Inhibition Assay

To verify the results obtained from the molecular docking calculations, an elastase inhibition assay was carried out, as reported previously [42]. Briefly, a 1 mM solution of the substrate (N-succinyl-Ala-Ala-Ala-p-nitroanilide) and a 10 μg/mL porcine pancreas elastase (PPE) solution were prepared in 0.1 M Tris-HCl buffer (pH 8). To a 96-well plate, 25 μL of the Tris-HCl buffer (0.1 M), 25 μL of PPE solution, and 25 μL of either the peptide (5000 μM) or a Tris-HCl solution (reference) were added. The plate was then incubated in the dark at 25 °C for 15 min, followed by the addition of 100 μL of the substrate solution or Tris-HCl buffer. The samples were incubated for another 15 min at 37 °C. After 30 min, absorbance was measured at 405 nm using the Infinite F/M 200 Pro Nanoquant plate reader (Tecan, Männedorf, Switzerland). The peptides were used at a concentration of 2.5 mmol/dm^3^.

Elastase inhibition (I_PPE_) was calculated using the following formula (1):

(1) $$I_{PPE}=\frac{\left(AR-AB\right)-(AP-AB)}{AR-AB}\cdot 100\%$$

where AR is the reference sample absorbance, AB is the blank absorbance, and AP is the peptide sample absorbance.

### 3.5. Preparation of Empty Liposomes and Peptide-Loaded Liposomes (PLLs)

Cationic liposomes (CLs) and anionic liposomes (ALs) were prepared using the thin-film hydration method described previously [42]. Briefly, all lipids, including hydrogenated lecithin (HL), cholesterol (ChL), and dicetyl phosphate (DCP) or stearylamine (SA), were dissolved in chloroform, and the solvent was evaporated on a rotary evaporator. The resulting lipid film was hydrated with phosphate-buffered saline (PBS) at pH 6 for empty liposomes or with a peptide solution in PBS (0.5 mg/cm^3^) for PLLs. The total lipid concentration was maintained at 25 mg/cm^3^ for all samples and the HL:ChL weight ratio was kept constant at 14:1. Five freeze–thaw cycles in liquid nitrogen were applied to the raw liposomes, which were then extruded 12 times through a polycarbonate membrane with 100 nm pores using an Avanti mini extruder (Avanti Polar Lipids Inc., Alabaster, AL, USA). Liposomes were stored at room temperature.

### 3.6. Characterization of PLLs

#### 3.6.1. Size Analysis and Zeta Potential

The hydrodynamic diameter and size distribution (polydispersity index, PDI) of the prepared vesicles were determined using dynamic light scattering (DLS) with a Malvern Zetasizer Nano ZS system (Malvern Panalytical Ltd., Malvern, UK). Zeta potential calculations were carried out using the Helmholtz–Smoluchowski equation. Measurements were carried out at 25 °C and repeated three times for each sample. All parameters were assessed over a 4-week period to evaluate liposome stability. During these stability tests, the liposomes were stored at room temperature (20–25 °C).

#### 3.6.2. Transmission Electron Microscopy (TEM)

TEM was used to further verify the size and shape of the liposomes. A 5 µL sample of the liposome dispersion was placed onto copper grids coated with a Formvar film. The measurements were carried out with a JEOL JEM 2100 HT transmission electron microscope (Jeol Ltd., Tokyo, Japan) operating at an accelerating voltage of 80 kV. Liposome images were captured with a 4k × 4k TVIPS camera, utilizing EMMENU software version 4.0.9.87 (TVIPS GmbH, Gauting, Germany).

#### 3.6.3. Encapsulation Efficiency (EE) and Loading Capacity (LC)

Peptide EE in the liposomes was determined via microdialysis. To separate the liposomes from the unencapsulated active ingredients, a regenerated cellulose membrane with a 6–8-kDa molecular weight cut-off (Spectra/Por) was used. The membrane was rinsed three times with distilled water and then immersed in PBS (pH 6) for 1 h. Liposome dispersion samples (1 cm^3^) were placed in the dialysis bag and transferred to a thermostatic chamber with PBS (100 cm^3^) for 4 h at room temperature (20–25 °C). The concentration of unencapsulated peptide that diffused into the solution was quantified by a DionexUltiMate 3000 HPLC system (Sunnyvale, CA, USA) with a DAD Thermo Scientific detector and a Kromasil 100-5-C18 column (Göteborg, Sweden). EE was calculated using Equation (2):

(2) $$EE=\frac{TP-UP}{TP}\cdot 100\%$$

where TP is the total peptide amount and UP is the unencapsulated peptide amount.

LC was calculated using Equation (3):

(3) $$LC=\frac{EP}{TLM}$$

where EP is the encapsulated peptide amount and TLM is the total mass of lipids used for the preparation of liposomes.

#### 3.6.4. Cytotoxicity

The cytotoxicity of the aqueous tripeptide solutions was determined in the previous work [35]. To assess the potential cytotoxicity of the synthesized peptides encapsulated within anionic liposomes, cell viability was tested using the EpiDerm-200 reconstructed human epidermis (RHE, MatTek Europe, Bratislava, Slovak Republic) model [72], following the procedure outlined in detail by Malinowska et al. [73]. Prior to the experiment, all RHE tissues underwent visual inspection to ensure they were free of any defects. The preparation involved sequential pre-incubation periods of 60 min and 18 h. Both incubations were carried out in a sterile environment at 37 °C with 95% relative humidity and a 5% CO_2_ atmosphere. The subsequent day, a 30 µL aliquot of the liposome dispersion was applied directly to the cells. The non-encapsulated peptide was not removed from the liposome samples. The concentration of the applied peptide was 0.5 mg/cm^3^, while the concentration of liposomes (lipids) was 25 mg/cm^3^. For controls, phosphate-buffered saline (PBS) at pH 7.4 served as the negative control, while a 5% solution of sodium dodecyl sulfate (SDS) was used as the positive control. The test compounds were in contact with the tissues for a duration of 60 min. Following this contact period, the tissues were thoroughly rinsed with PBS to remove residual test substances and transferred to fresh culture medium. This medium was replaced again after a 24 h incubation period. The total incubation and recovery time before assessment was 42 h. After this period, cell viability was determined using the MTT assay. Tissues were transferred to a 3-(4,5-dimethylthiazol-2-yl)-2,5-diphenyltetrazolium bromide (MTT) solution and incubated for 3 h. The resulting formazan product was then extracted by transferring samples to a fresh plate and agitating them in isopropanol at 120 rpm for 2 h. The absorbance of the extracted formazan was measured at 570 nm using a UV–visible spectrophotometer (Nanocolor™; Macherey-Nagel, Düren, Germany), with isopropanol used as the blank.

Relative cell viability (RCV) was then calculated according to Equation (4):

(4) $$RCV=\frac{A_{TS}}{A_{NC}}\cdot 100\%$$

where A_TS_ is the test sample absorbance and A_NC_ is the negative control absorbance.

### 3.7. Permeation Studies

For permeation studies, the synthetic membrane Strat-M^®^ and skin samples were used. The methodology was the same for both assays. The CSF tripeptide was selected for testing, given that it exhibited the highest tyrosinase inhibitory activity among the synthesized compounds [35]. CSF-AL liposomes were used for penetration experiments. Additionally, the peptide dissolved in a 50% aqueous solution of Transcutol^®^ P (a known enhancer of transdermal penetration) was used for comparison. A donor compartment replicate with PBS was used for analytical purposes.

Permeation tests of the CSF tripeptide were performed using a Franz cell apparatus (MicroettePlus^®^ system, Hanson Research, CA, USA) with Strat-M^®^ and/or abdominal human skin (0.4 mm thickness, Biopredic Int.). Biopredic International holds a permit (AC-2013-1754) granted by the French Ministry of Higher Education and Research, authorizing the acquisition, processing, sale, and export of human biological material for research purposes. The cell setup was characterized by a diffusion surface area of 1.77 cm^2^ and a receptor volume of 7 mL. Prior to testing, transepidermal water loss (TEWL) measurements (Dermalab^®^, Cortex Technology, Hadsund, Denmark) were carried out to assess the barrier integrity (Table S1). A sample volume of 0.5 mL of each test formulation, containing CSF at 0.5 mg/mL, was applied to the donor compartment facing the stratum corneum (SC). PBS (pH 7.4), maintained at 32 ± 1 °C, served as the receptor medium, with the solution stirred by a magnetic bar at 450 rpm. Over a 24 h period, 0.8 mL samples were periodically collected from the receptor compartment, with an equal volume of PBS replenished to maintain the total 7 mL volume. The permeated peptide amount was measured in triplicate by high-performance liquid chromatography (HPLC) on a Shimadzu CBM-20A system (Shimadzu, Kyoto, Japan) with a UV detector.

The skin samples remaining after the penetration tests were subsequently subjected to extraction to determine the amount of tripeptide retained in the skin. For this purpose, the skin samples were rinsed three times with 0.6 mL of a 0.25% Tween 80 solution in PBS, followed by two rinses with 0.6 mL PBS. The skin was then cut into small pieces, placed in a vial, and immersed in 2 mL of PBS. This setup was mixed using a magnetic stirrer (900 rpm) for 24 h. After extraction, the liquid was filtered from the solid skin residues using syringe filters (Millex, Merck, Darmstadt, Germany) with a pore size of 0.45 µm. The peptide concentration was determined by HPLC.

## 4. Conclusions

In this study, tripeptides (CSF, CVL, and CSN) with tyrosinase-inhibiting properties were synthesized and characterized. Complementing their skin-lightening capabilities, these compounds also demonstrated the ability to inhibit elastase activity, and the underlying mechanism was confirmed through molecular modeling. Liposomes containing the tested peptides exhibited small, uniform diameters of approximately 100 nm. The EE across all carriers was comparable, likely due to the similar chemical structures of the peptides. Cytotoxicity tests on RHE cells confirmed the safety of the prepared liposomal formulations, as all samples demonstrated no cytotoxic effects. In percutaneous penetration tests, none of the peptides were detected in the acceptor solution; however, both CSF and GHK peptides showed significant absorption in the skin. This localized retention property is beneficial for anti-aging and skin-brightening applications, as it ensures delivery to the target site without systemic absorption. In conclusion, the synthesized bioactive peptides and their liposomal carriers show great promise as versatile raw materials for use in dermocosmetic formulations. Collectively, the confirmed safety and targeted action of these peptides offer valuable resources to the field of dermatological research and product development.

## Figures and Tables

**Figure 1:**
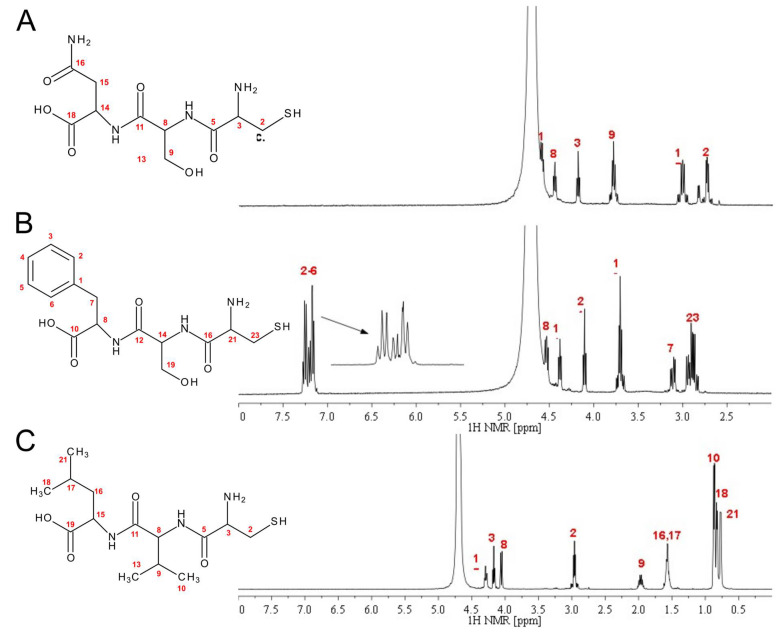
The comparison of the three ^1^H NMR spectra with amino acid assignments for the CSN (**A**), CSF (**B**), and CVL (**C**) tripeptides.

**Table 1:** In vitro (inhibition activity) and in silico (docking score) results of the elastase inhibitory assay for the studied tripeptides. Values are displayed as ±standard deviation (n = 3).

Peptide	Inhibition Activity [%] ± SD	Docking Score [kcal/mol]
CSF	27.6 ± 1.2	−6.149
CVL	35.3 ± 8.6	−4.755
CSN	28.9 ± 7.5	−5.304

**Table 2:** Zeta potential, encapsulation efficiency (EE), and loading capacity (LC) of cationic liposomes (CL, 7% SA) and anionic liposomes (AL, 15% DCP) containing the synthesized tripeptides CSF, CVL, and CSN. The total lipid concentration was constant at 25 mg/cm^3^ for all liposome samples.

Liposomes	Zeta Potential ± SD [mV]	EE ± SD [%]	LC ± SD [(mg of Peptide)/(mg of Total Lipids)]
CL	42.7 ± 2.5	-	-
CSF-CL	34.2 ± 1.5	33.98 ± 6.90	0.68 ± 0.14
CVL-CL	36.0 ± 1.9	25.98 ± 9.58	0.52 ± 0.19
CSN-CL	44.7 ± 2.6	28.99 ± 5.67	0.58 ± 0.11
AL	−38.6 ± 1.3	-	-
CSF-AL	−38.7 ± 2.2	29.65 ± 6.91	0.59 ± 0.14
CVL-AL	−37.4 ± 1.3	21.01 ± 2.49	0.42 ± 0.05
CSN-AL	−35.1 ± 6.4	26.71 ± 4.08	0.53 ± 0.08

## Data Availability

Data is contained within the article and Supplementary Materials.

## Supplementary Materials

The following supporting information can be downloaded at: https://www.mdpi.com/article/10.3390/ijms26115321/s1**.**

## Abbreviations

AL, anionic liposomes; CL, cationic liposomes; DLS, dynamic light scattering; EE, encapsulation efficiency; HL, hydrogenated lecithin; HPLC, high-performance liquid chromatography; LUV, large unilamellar vesicles; MWCO, molecular weight cut-off; NC, negative control; NMR, nuclear magnetic resonance; PBS, phosphate-buffered saline; PC, positive control; PLL, peptide-loaded liposomes; PPE, porcine pancreas elastase; RCV, relative cell viability; RHE, reconstructed human epidermis; RV, relative viability; SC, stratum corneum; TEM, transmission electron microscopy; TEWL, transepidermal water loss; UV, ultraviolet–visible.
