# Supplementary material for: Tripeptide-Loaded Liposomes as Multifunctional Components in Topical Formulations
Source: Int J Mol Sci. 2025 Jun 1;26(11):5321. doi: 10.3390/ijms26115321 (PMC12154373; doi:10.3390/ijms26115321)
Supplement: Supplementary file 1 [file ijms-26-05321-s001.zip › ijms-3578536-SM.pdf]

International Journal of *Molecular Sciences*

MDPI

Supplementary Materials

Tripeptide-Loaded Liposomes as Multifunctional Components in Topical Formulations

Michał Dymek[1,*], Maria José García-Celma[2], Elvira Escribano-Ferrer[3], Dawid Warszycki[4],Sławomir Kaźmierski[5], Łukasz Skoczylas[6], Małgorzata Tabaszewska[6,7] and Elżbieta Sikora[8,*]

[1] Cracow University of Technology, CUT Doctoral School, Faculty of Chemical Engineering and Technology, 31-155 Kraków, Poland
[2] University of Barcelona, Department of Pharmacy, Pharmaceutical Technology and Physical-Chemistry, Pharmaceutical Nanotechnology Group, E-08028 Barcelona, Spain
[3] University of Barcelona, Faculty of Pharmacy and Food Sciences, Department of Pharmacy and Pharmaceutical Technology and Physical Chemistry, Biopharmaceutics and Pharmacokinetics Unit, E-08028 Barcelona, Spain
[4] Polish Academy of Sciences, Maj Institute of Pharmacology, Department of Medicinal Chemistry, 31-343 Kraków, Poland
[5] Polish Academy of Sciences, Centre of Molecular and Macromolecular Studies, 90-363 Łódź, Poland
[6] University of Agriculture in Kraków, Faculty of Food Technology, Department of Plant Product Technology and Nutrition Hygiene, 30-149 Kraków, Poland.
[7] Department of Human Nutrition and Metabolomics, Pomeranian Medical University in Szczecin, 71-460 Szczecin, Poland.
[8] Cracow University of Technology, Faculty of Chemical Engineering and Technology, 31-155 Kraków, Poland

* Correspondence: michal.dymek@doktorant.pk.edu.pl (M.D.) and elzbieta.sikora@pk.edu.pl (E.S.)

International Journal of Molecular Sciences

MDPI

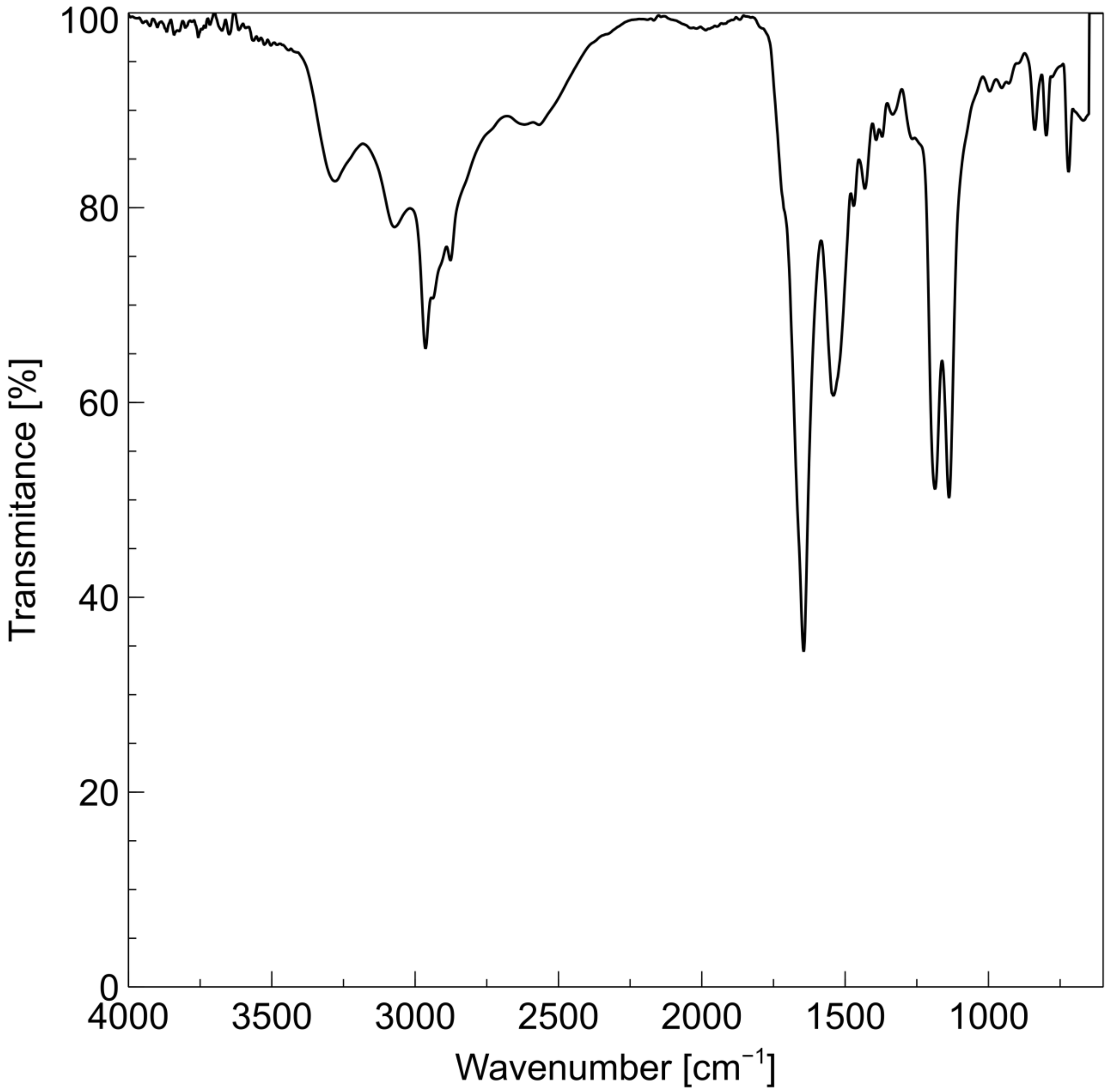

Figure S1. IR spectrum of CVL tripeptide.

International Journal of
Molecular Sciences

MDPI

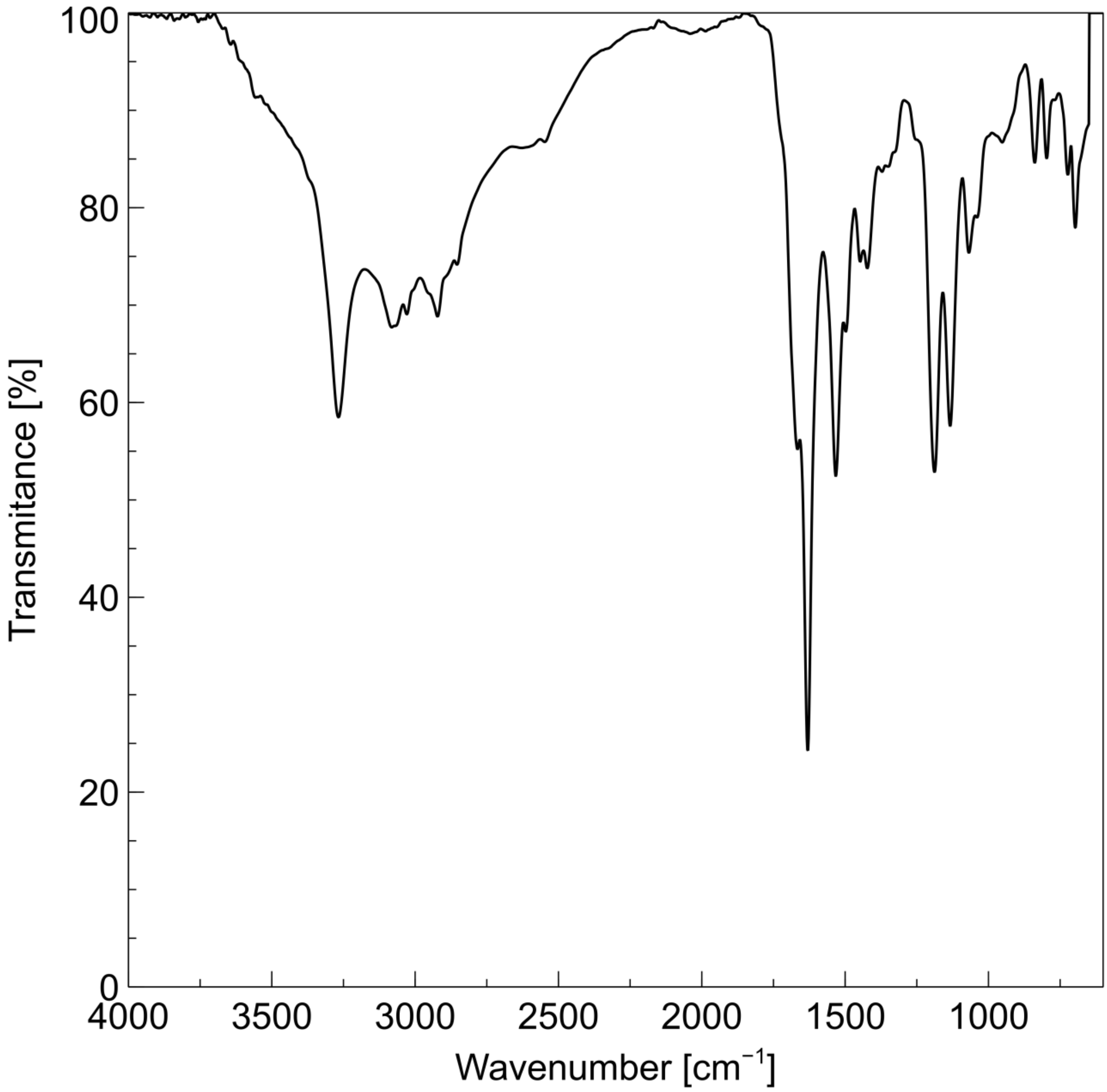

Figure S2. IR spectrum of CSF tripeptide.

International Journal of *Molecular Sciences*

MDPI

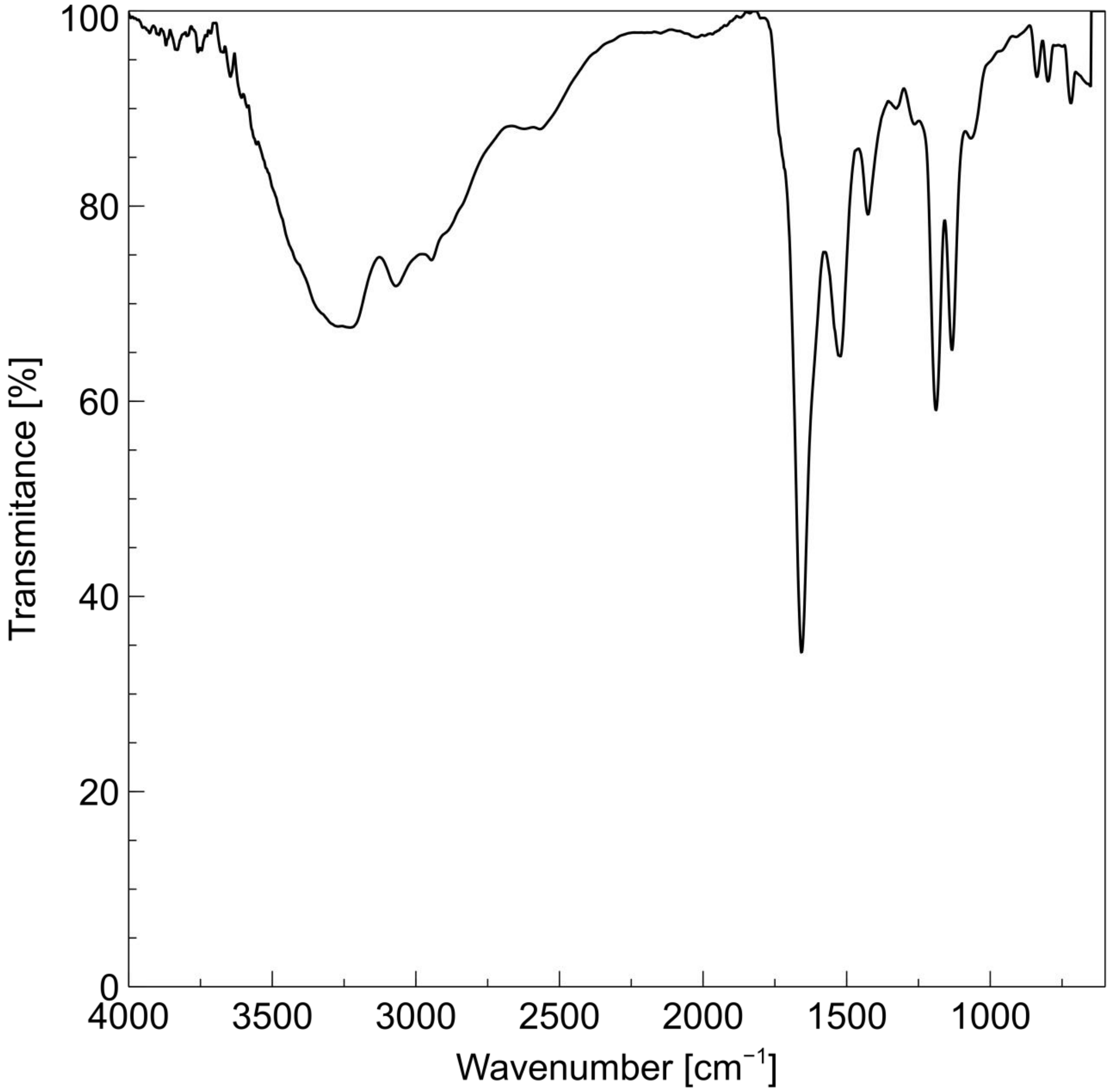

Figure S3. IR spectrum of CSN tripeptide.

International Journal of
Molecular Sciences

MDPI

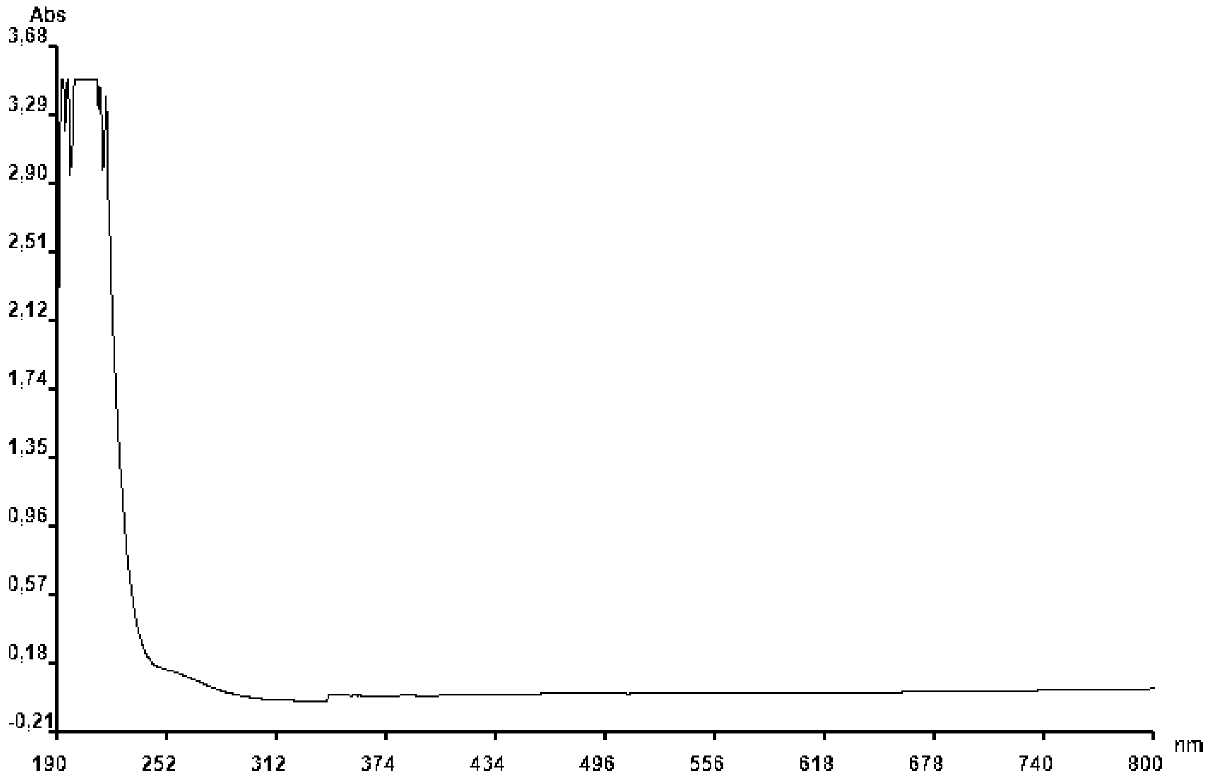

Figure S4. UV-Vis spectra of the CVL tripeptide.

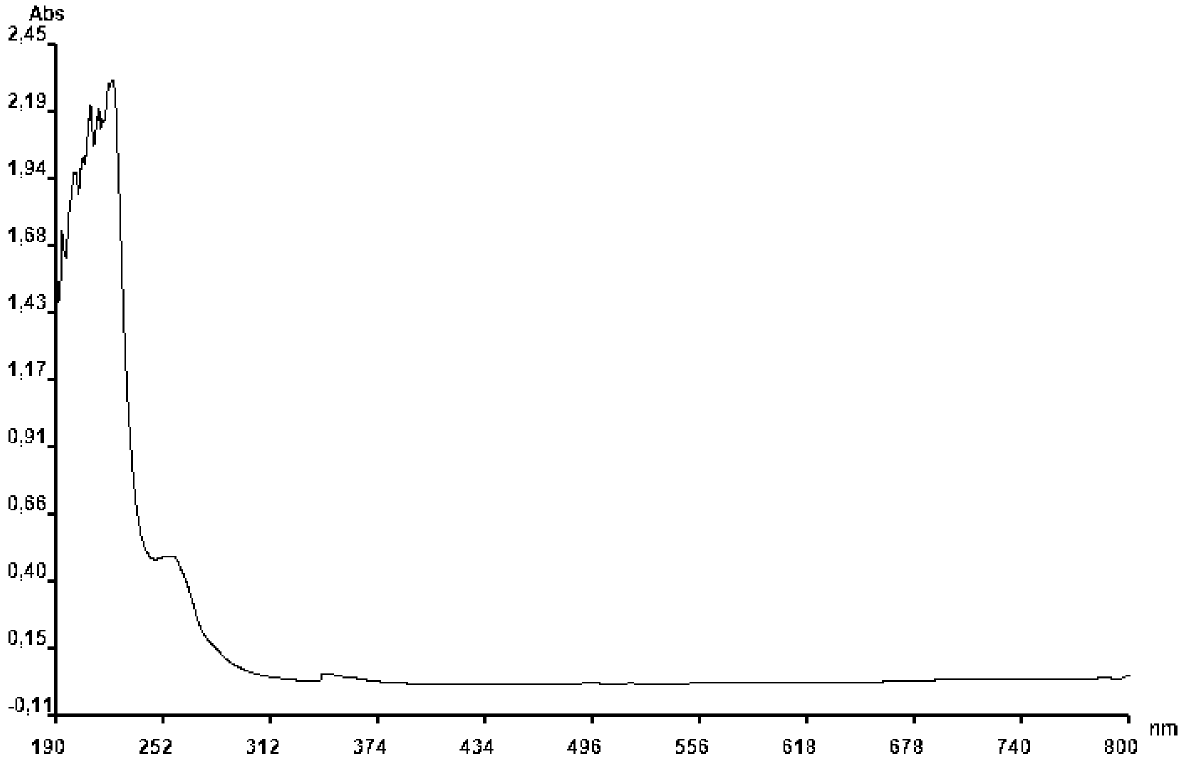

Figure S5. UV-Vis spectra of the CVL tripeptide.

International Journal of
Molecular Sciences

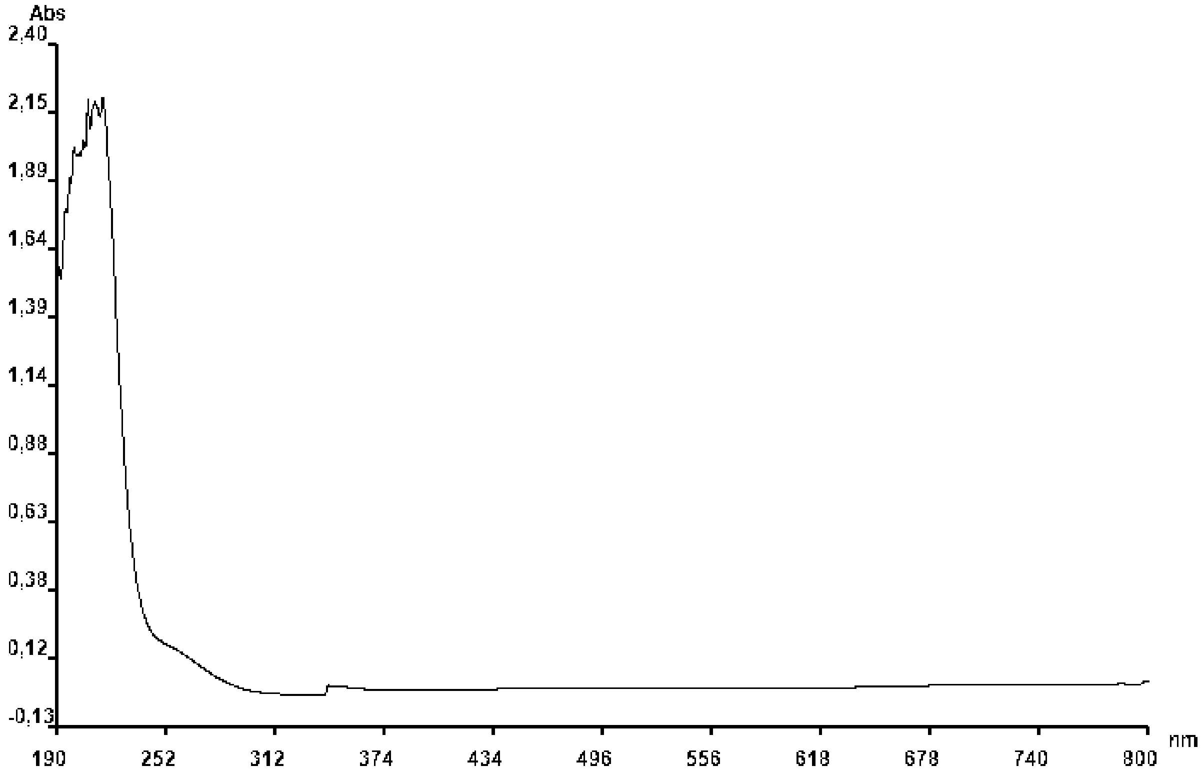

Figure S6. UV-Vis spectra of the CSN tripeptide.

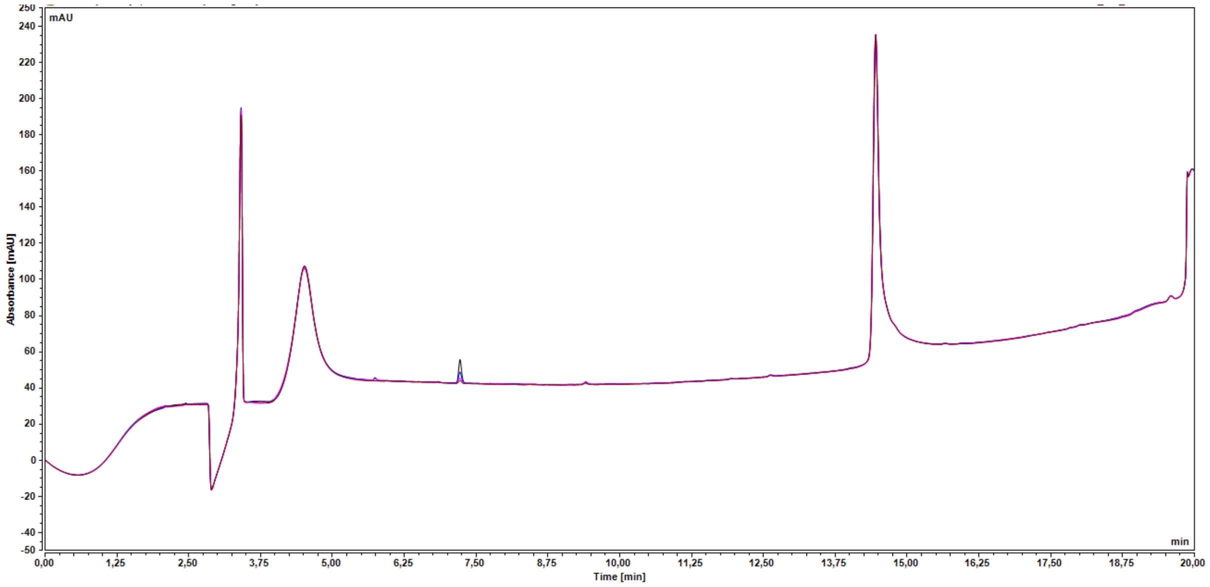

Figure S7. Chromatogram of the CVL tripeptide. Retention time, RT_{CVL} = 7.225 min.

International Journal of Molecular Sciences

MDPI

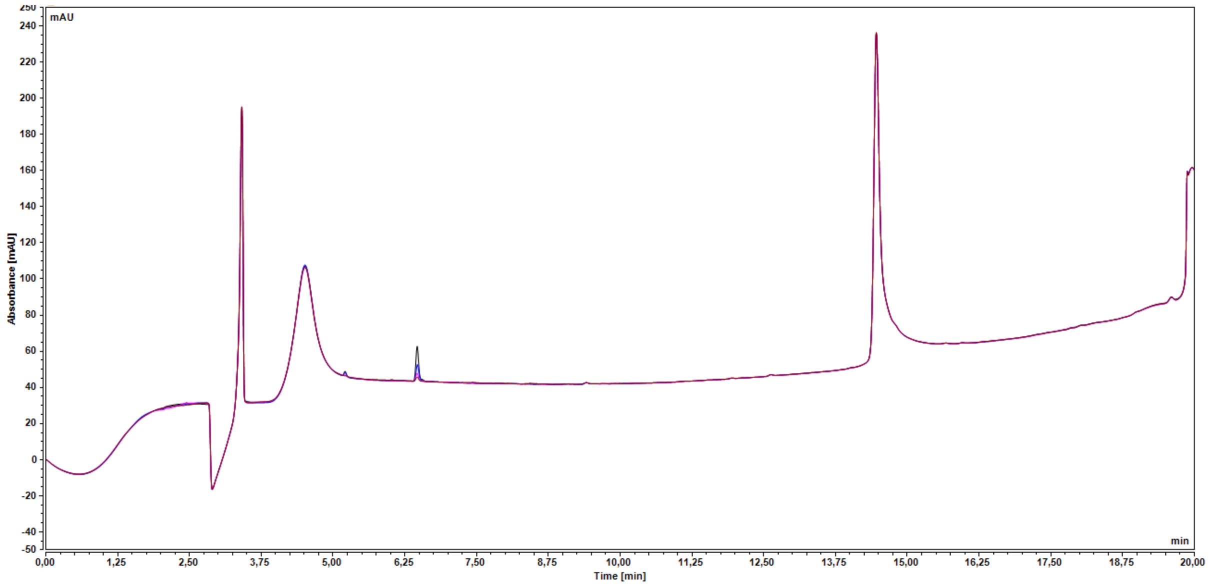

Figure S8. Chromatogram of the CSF tripeptide. Retention time, RT_{CSF} = 6.475 min.

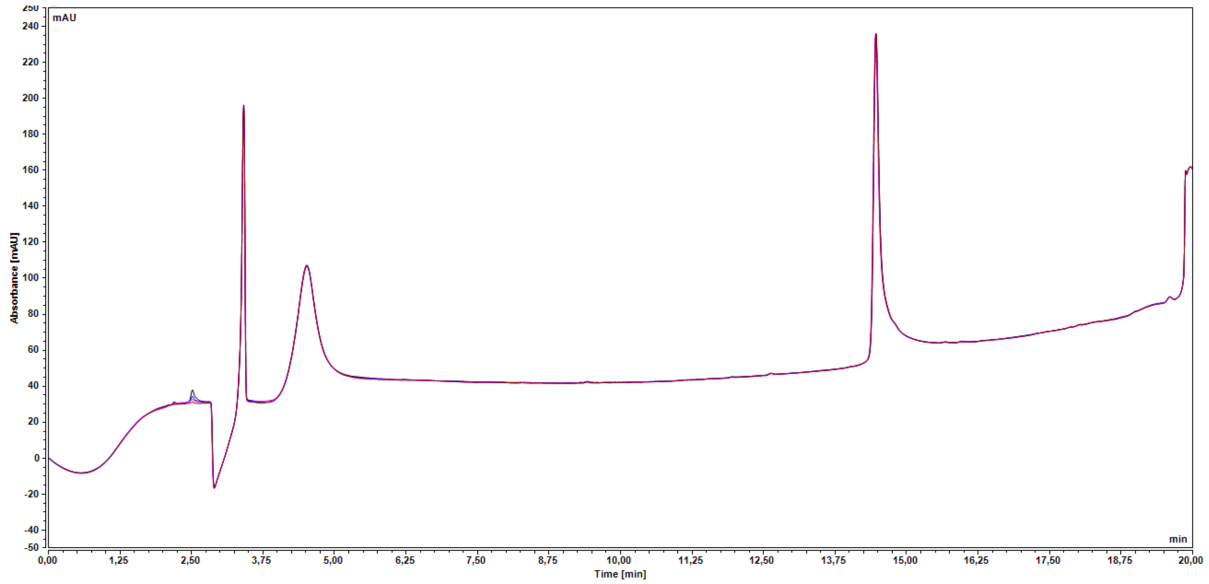

Figure S9. Chromatogram of the CSN tripeptide. Retention time, RT_{CSN} = 2.525 min.

International Journal of
Molecular Sciences

MDPI

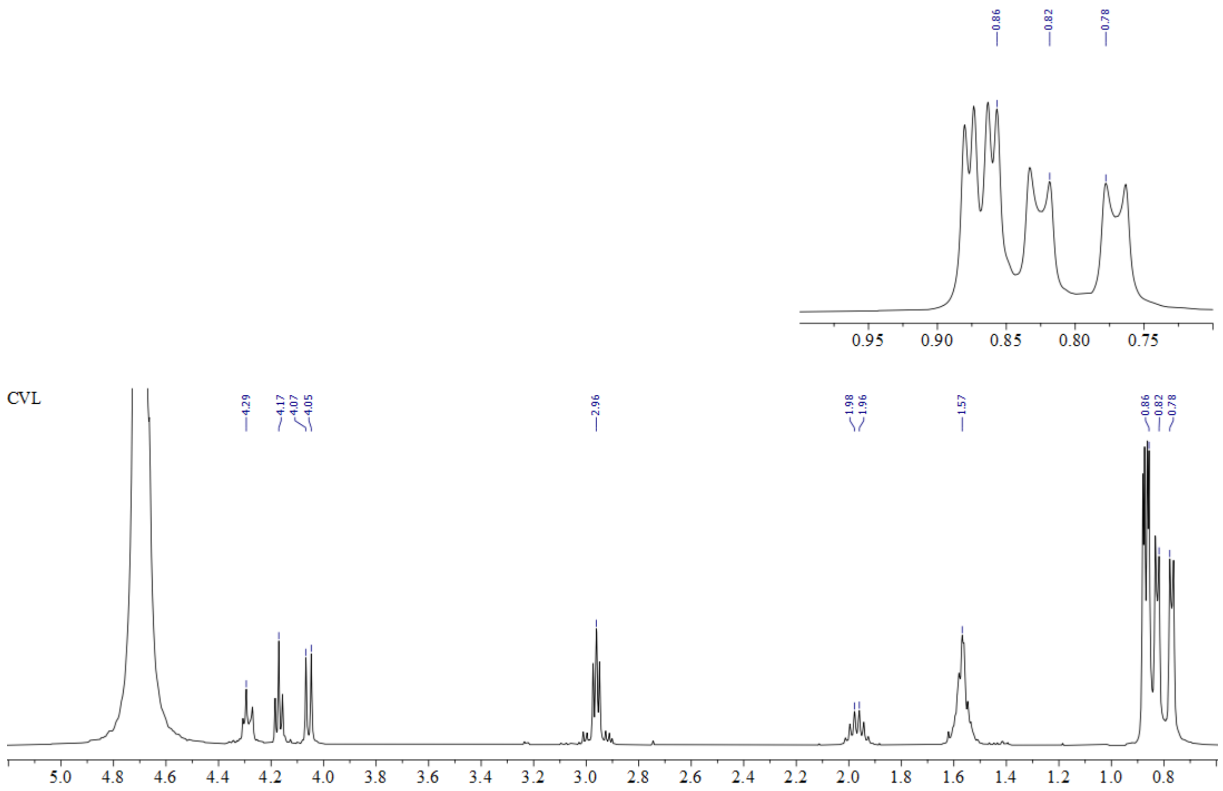

Figure S10. ^{1}H-NMR spectrum of the CVL tripeptide.

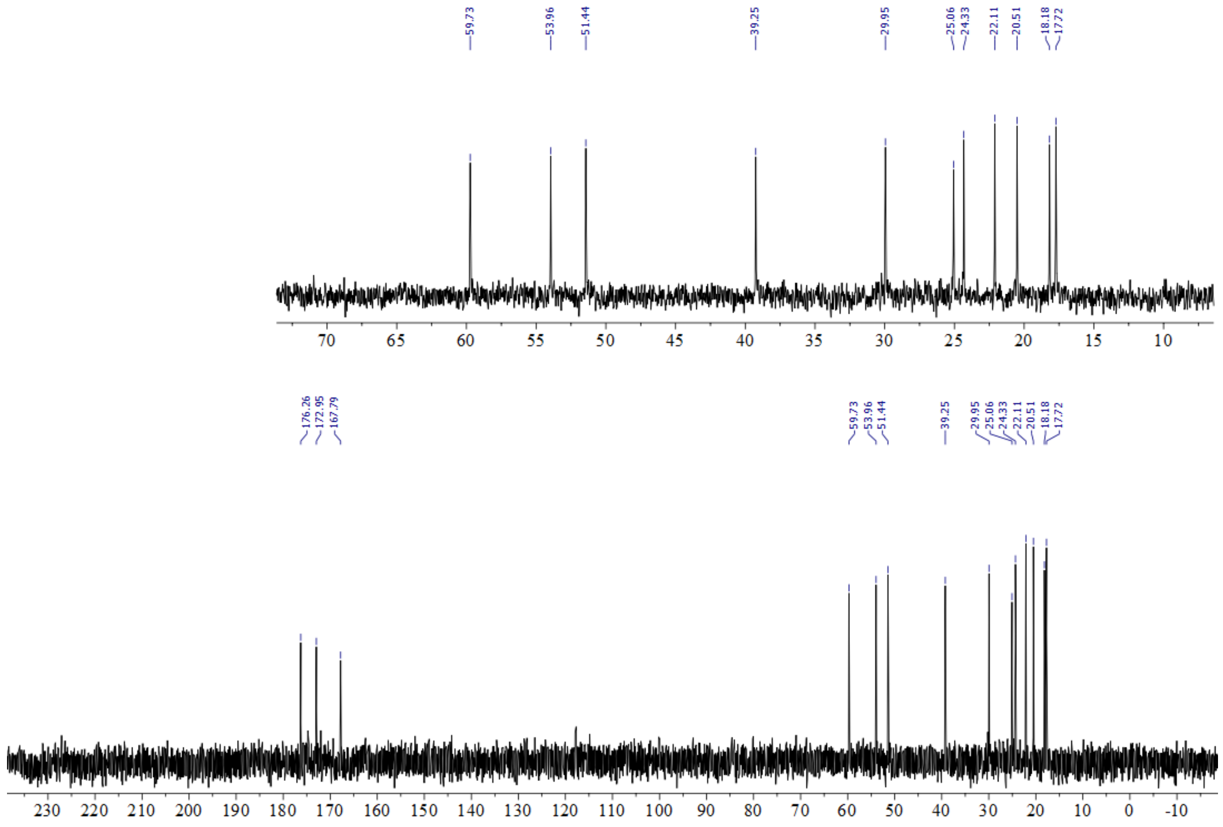

Figure S11. ^{13}C-NMR spectrum of the CVL tripeptide.

International Journal of
Molecular Sciences

MDPI

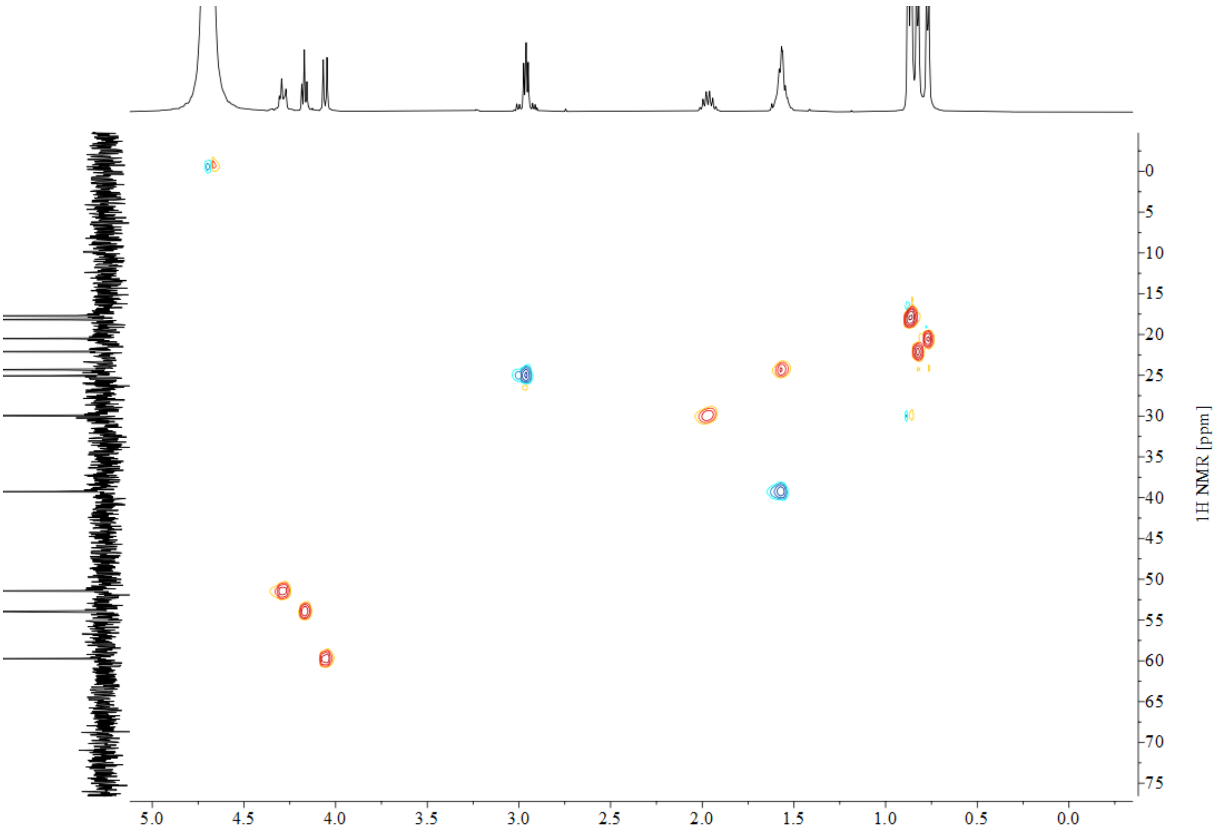

Figure S12. ^{1}H–^{13}C HSQC spectra of the CVL tripeptide.

International Journal of Molecular Sciences

MDPI

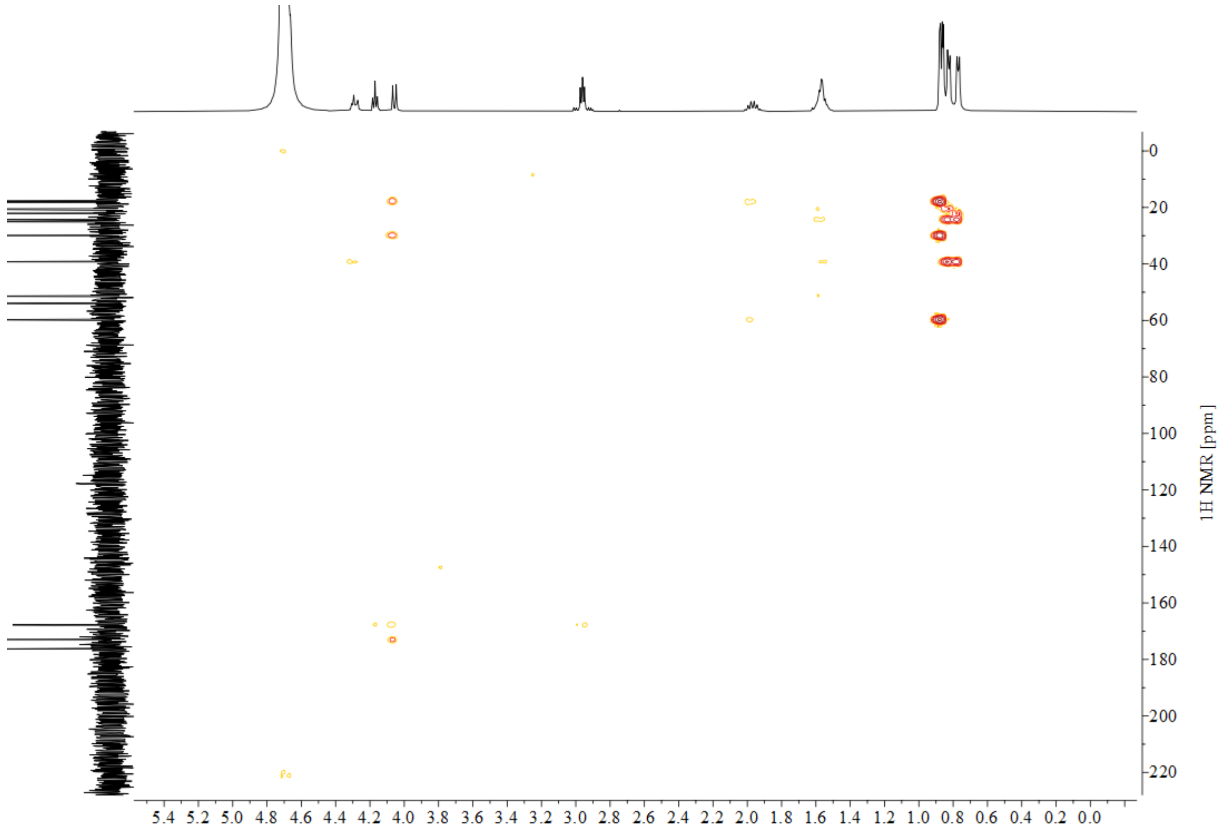

Figure S13. ^{1}H–^{13}C HMBC spectra of the CVL tripeptide.

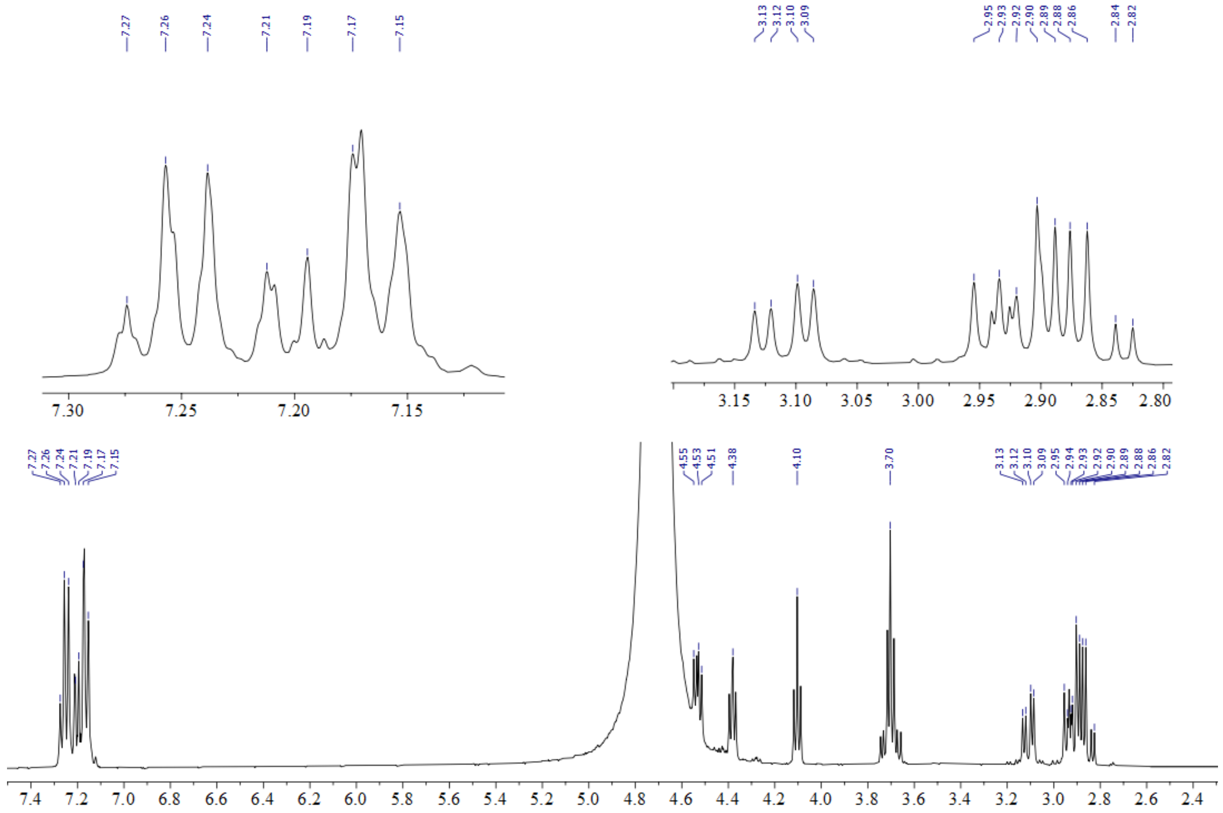

Figure S14. ¹H-NMR spectrum of the CSF tripeptide.

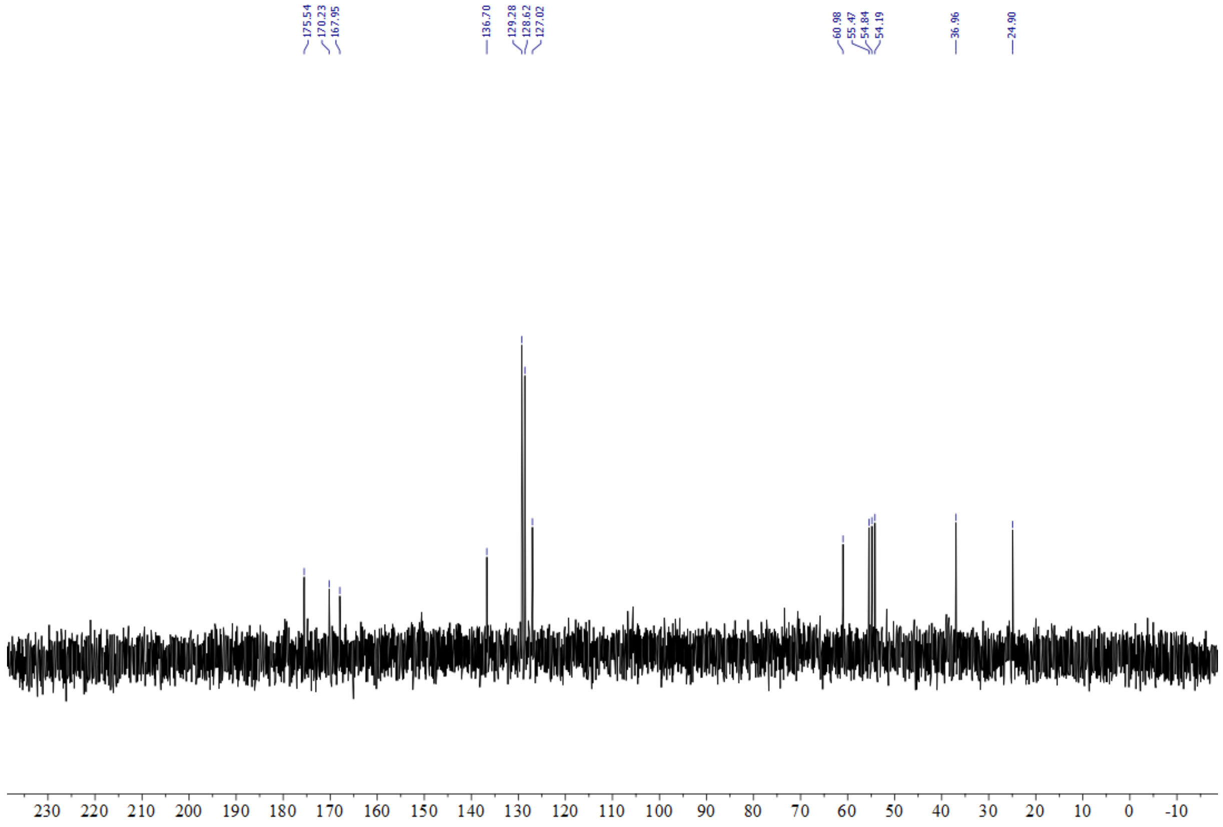

Figure S15. ¹³C-NMR spectrum of the CSF tripeptide.

International Journal of Molecular Sciences

MDPI

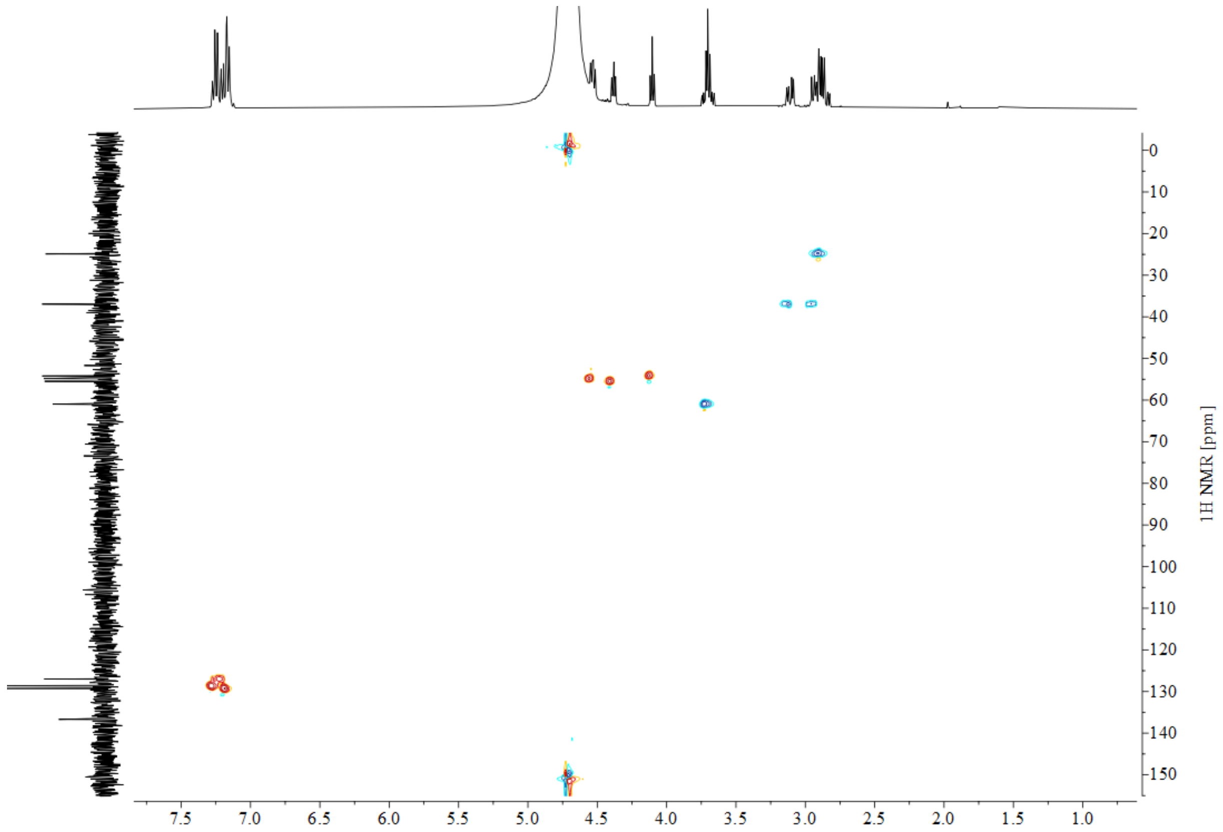

Figure S16. ^{1}H–^{13}C HSQC spectra of the CSF tripeptide.

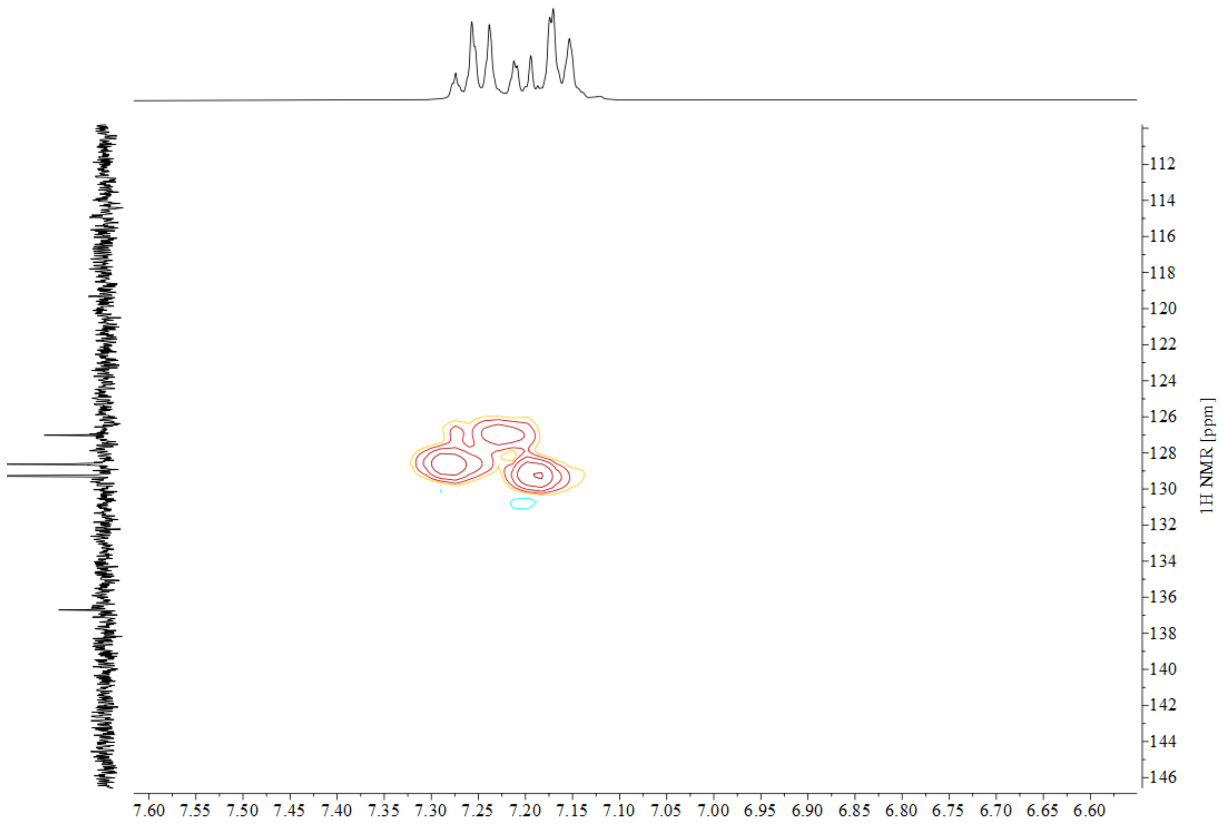

Figure S17. ^1H–^{13}C HSQC detailed spectra of the CSF tripeptide.

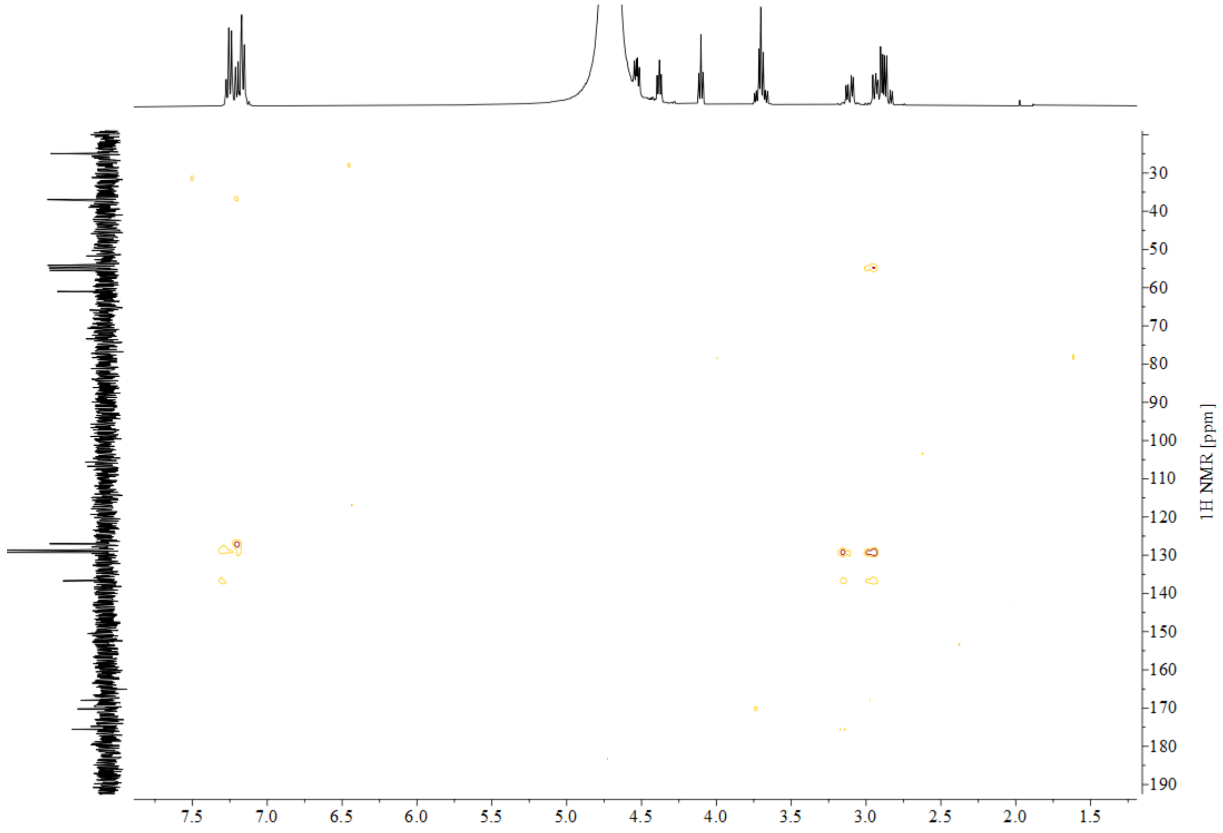

International Journal of
Molecular Sciences

MDPI

Figure S18. ^{1}H–^{13}C HMBC spectra of the CSF tripeptide.

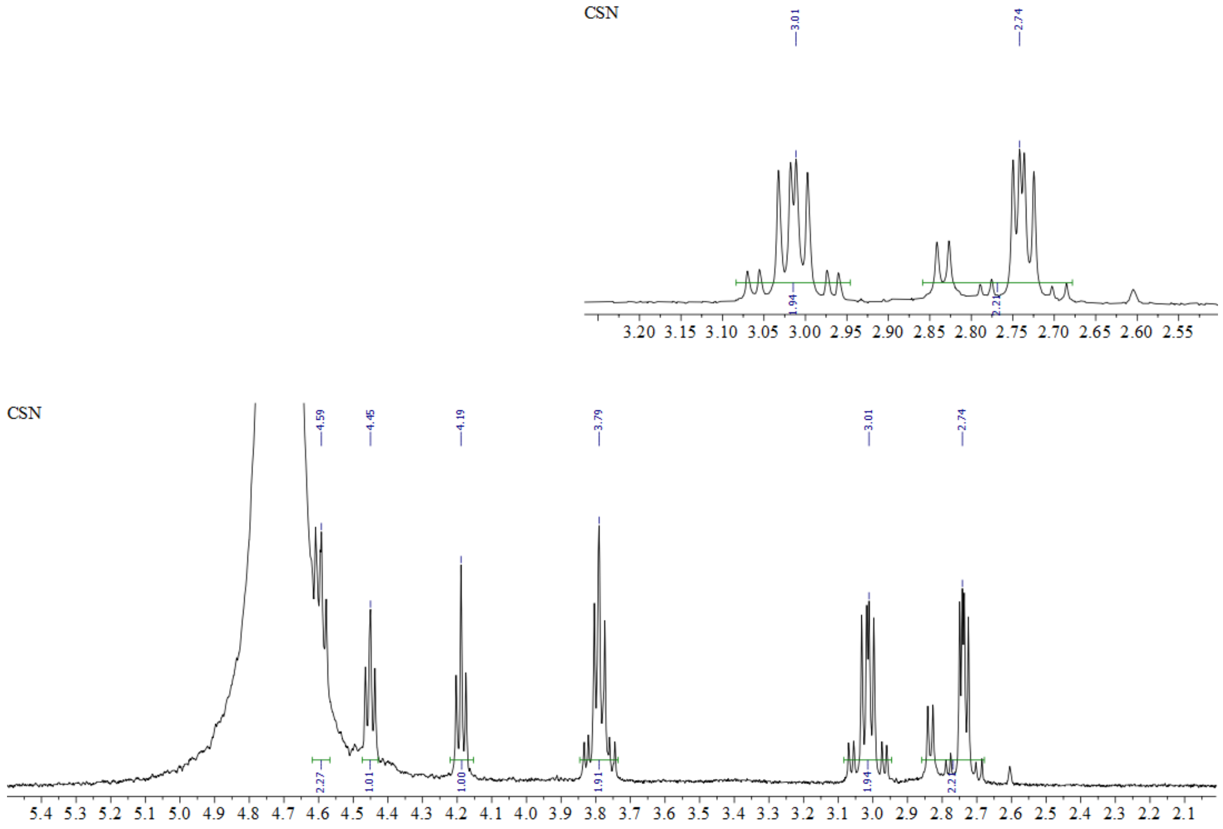

Figure S19. ^{1}H-NMR spectrum of the CSN tripeptide.

International Journal of
Molecular Sciences

MDPI

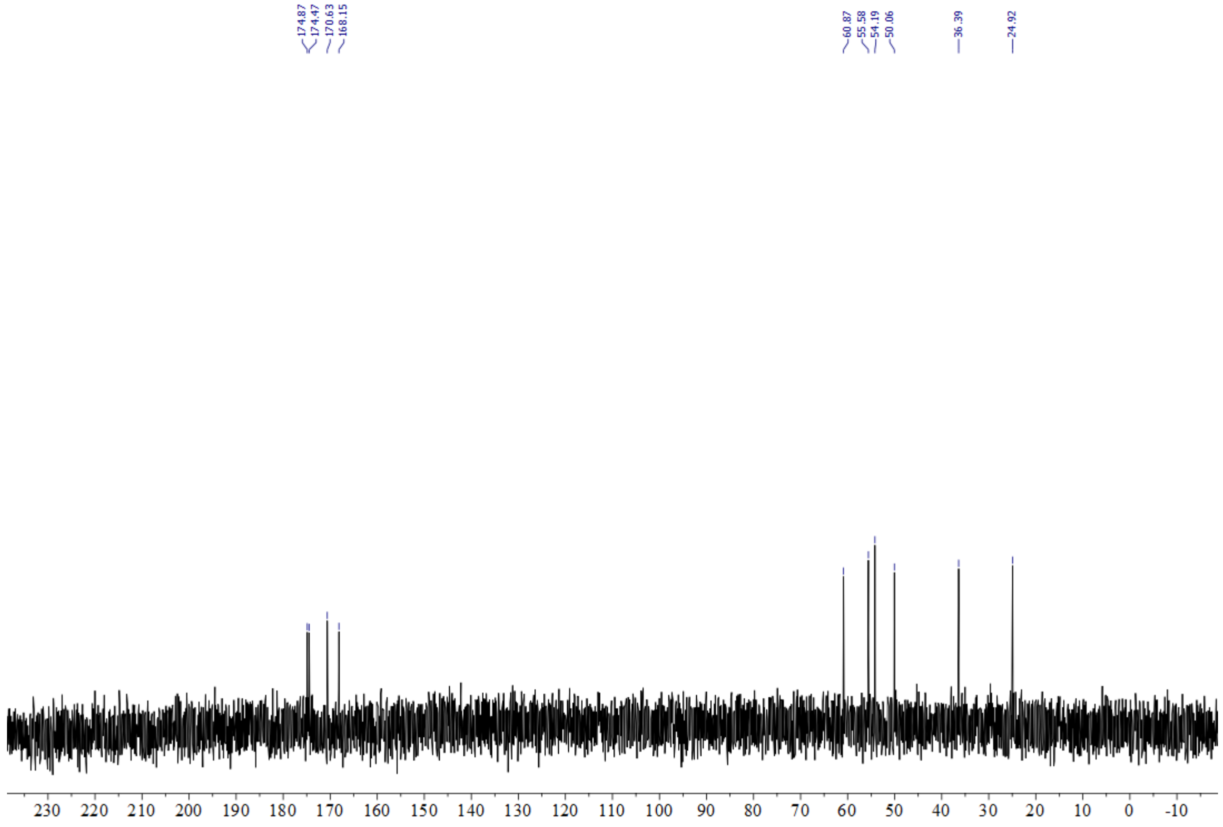

Figure S20. ^{13}C-NMR spectrum of the CSN tripeptide.

International Journal of
Molecular Sciences

MDPI

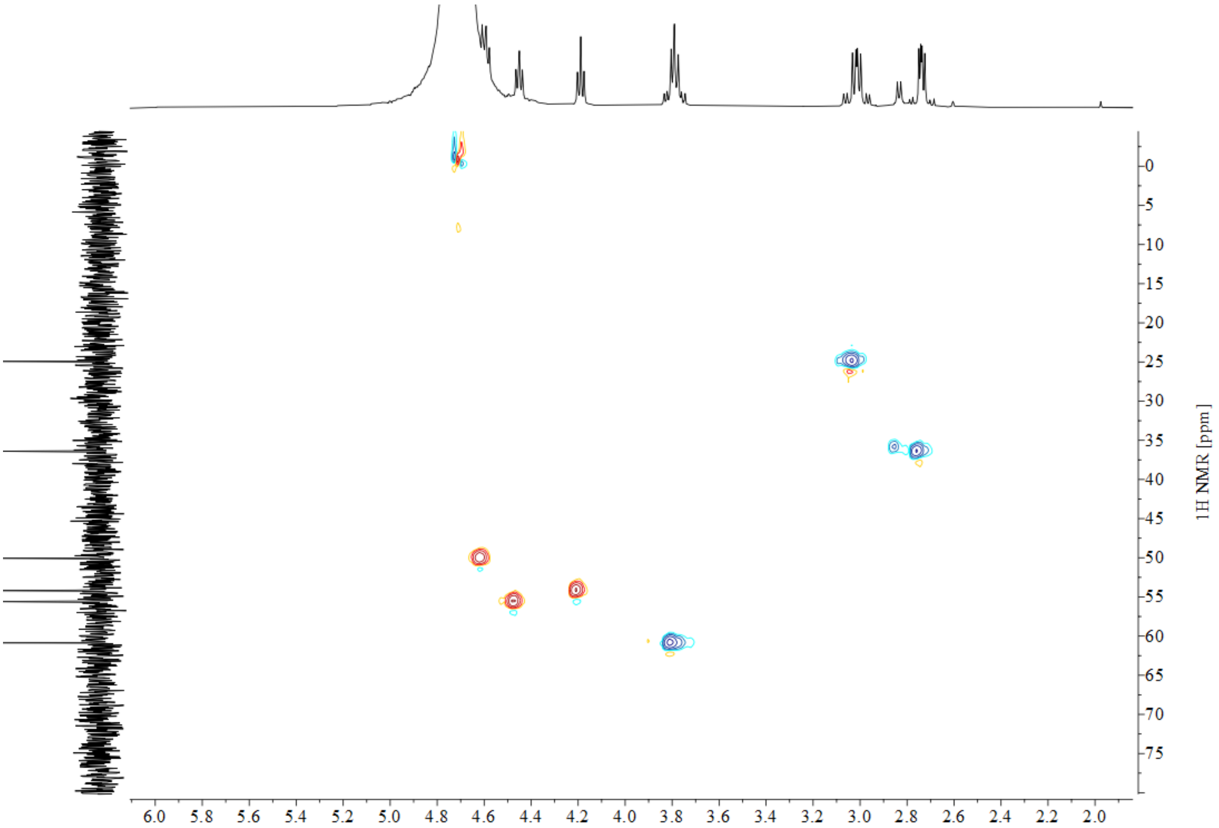

Figure S21. ^{1}H–^{13}C HSQC spectra of the CSN tripeptide.

International Journal of
Molecular Sciences

MDPI

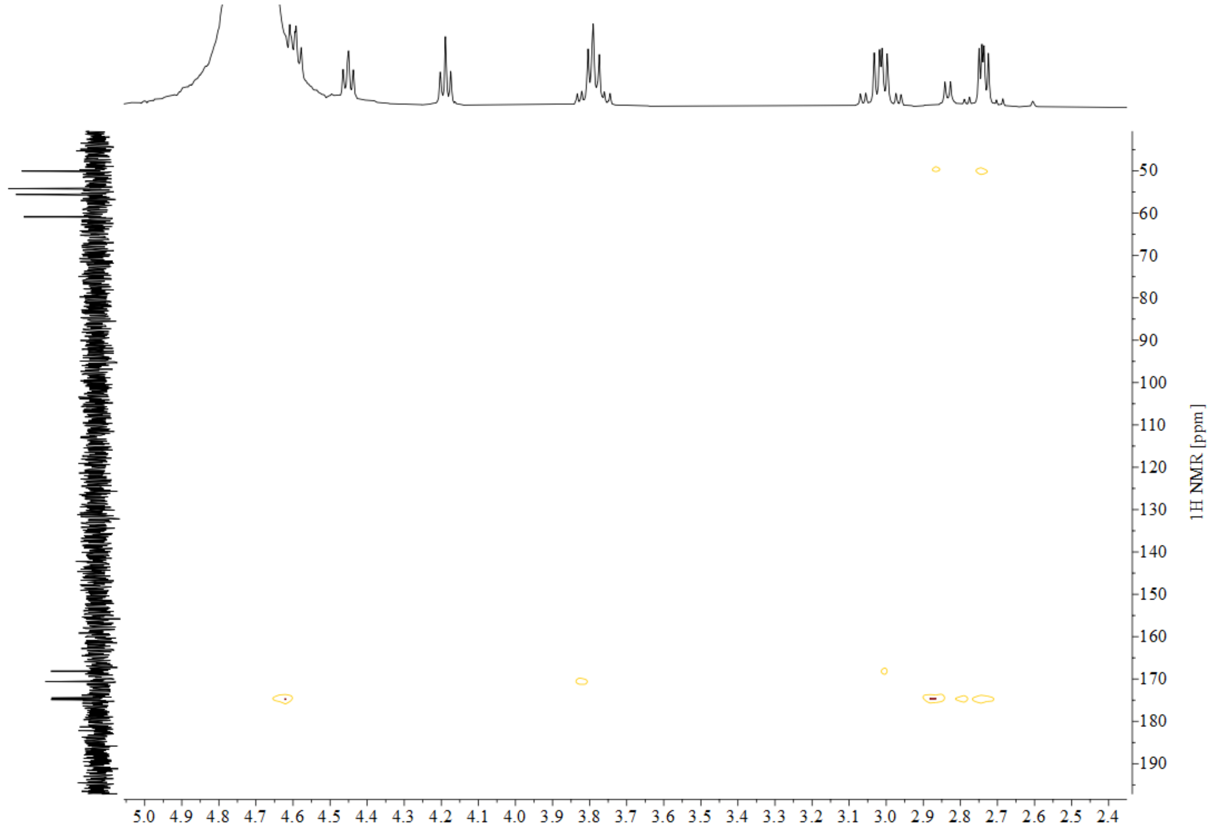

Figure S22. ^{1}H–^{13}C HMBC spectra of the CSN tripeptide.

International Journal of Molecular Sciences

MDPI

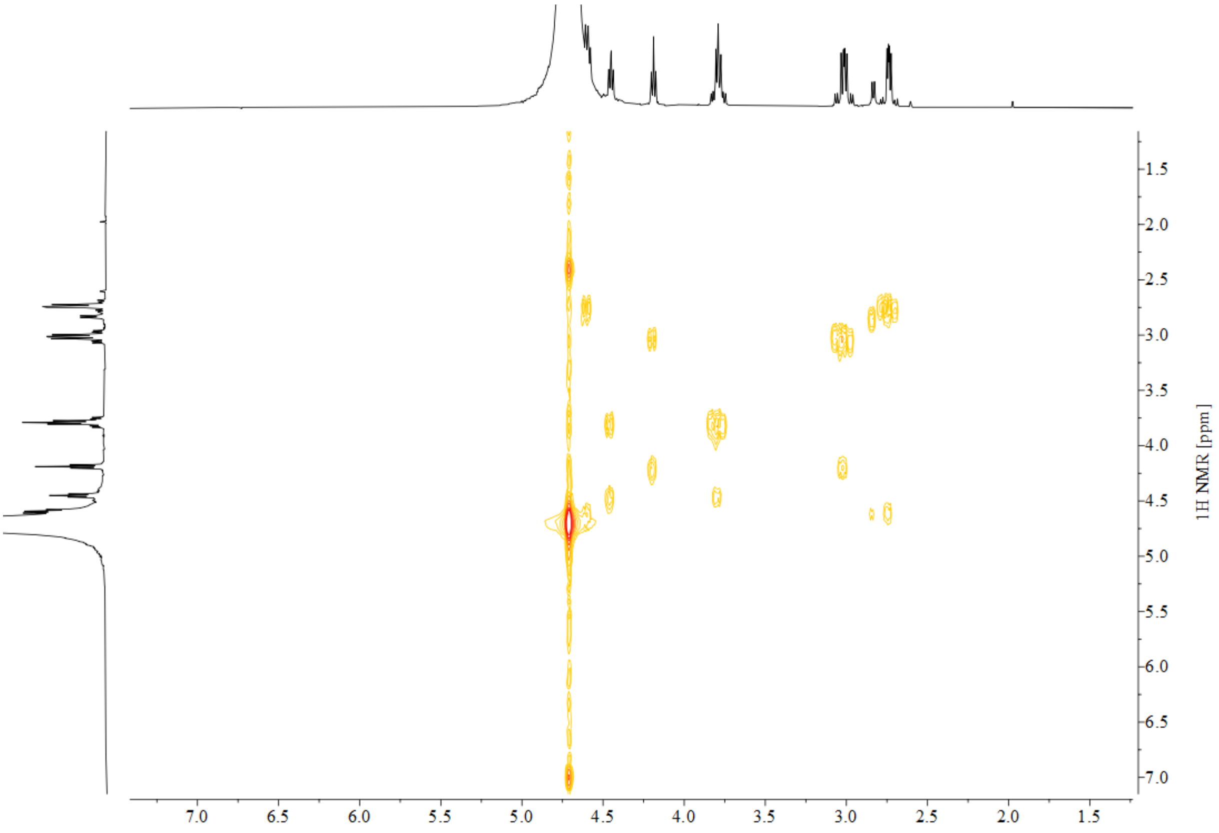

Figure S23. ^1H–^1H COSY spectra of the CSN tripeptide.

International Journal of Molecular Sciences

MDPI

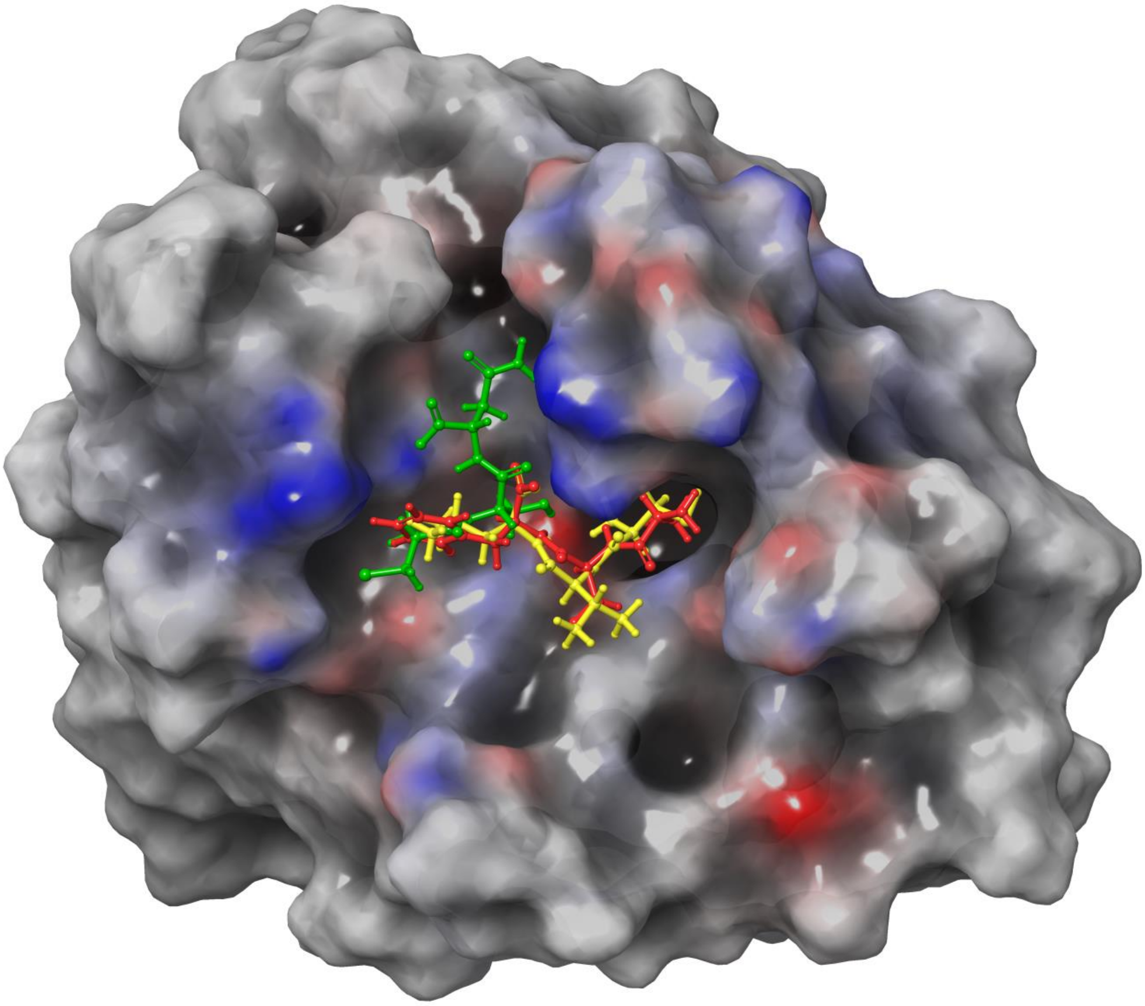

Figure S24. The superimposed docking poses of the CVL (yellow), CSF (red), and CSN (green) tripeptides within the elastase (1BRU) binding site. The surface of the enzyme is colored by electrostatic potential, with blue indicating more positive and red indicating more negative regions. The CSF and CVL peptides exhibit similar orientations on the enzyme surface, whereas CSN positions its N-terminus in a region of higher positive electrostatic potential.

Table S25. Assessment of the barrier integrity – transepidermal water loss (TEWL) measurements.

**Experiment**	**TEWL [g · m^{-2} · h^{-1}]**					
	**Franz cell position**					
	1	2	3	4	5	6
I	4.7	4.2	4.3	4.4	4.0	4.0
II	5.6	5.5	4.2	5.0	5.1	4.1
III	4.8	4.4	4.8	4.3	4.6	4.5
